# Auxiliary Screening COVID-19 by Serology

**DOI:** 10.3389/fpubh.2022.819841

**Published:** 2022-08-02

**Authors:** Xiongfeng Pan, Atipatsa C. Kaminga, Yuyao Chen, Hongying Liu, Shi Wu Wen, Yingjing Fang, Peng Jia, Aizhong Liu

**Affiliations:** ^1^Department of Epidemiology and Health Statistics, Xiangya School of Public Health, Central South University, Changsha, China; ^2^Department of Mathematics and Statistics, Mzuzu University, Mzuzu, Malawi; ^3^OMNI Research Group, Ottawa Hospital Research Institute, Ottawa, ON, Canada; ^4^Department of Obstetrics and Gynaecology, University of Ottawa Faculty of Medicine, Ottawa, ON, Canada; ^5^School of Epidemiology and Public Health, University of Ottawa Faculty of Medicine, Ottawa, ON, Canada; ^6^School of Resource and Environmental Sciences, Wuhan University, Wuhan, China; ^7^International Institute of Spatial Lifecourse Health (ISLE), Wuhan University, Wuhan, China

**Keywords:** COVID-19, serum specific antibody, novel coronavirus, meta-analysis, nucleic acid detection

## Abstract

**Background:**

The 2019 novel coronavirus (COVID-19) pandemic remains rampant in many countries/regions. Improving the positive detection rate of COVID-19 infection is an important measure for control and prevention of this pandemic. This meta-analysis aims to systematically summarize the current characteristics of the auxiliary screening methods by serology for COVID-19 infection in real world.

**Methods:**

Web of Science, Cochrane Library, Embase, PubMed, CNKI, and Wangfang databases were searched for relevant articles published prior to May 1^st^, 2022. Data on specificity, sensitivity, positive/negative likelihood ratio, area under curve (AUC), and diagnostic odds ratio (dOR) were calculated purposefully.

**Results:**

Sixty-two studies were included with 35,775 participants in the meta-analysis. Among these studies, the pooled estimates for area under the summary receiver operator characteristic of IgG and IgM to predicting COVID-19 diagnosis were 0.974 and 0.928, respectively. The IgG dOR was 209.78 (95% CI: 106.12 to 414.67). The IgM dOR was 78.17 (95% CI: 36.76 to 166.25).

**Conclusion:**

Our findings support serum-specific antibody detection may be the main auxiliary screening methods for COVID-19 infection in real world.

## Introduction

Three unprecedented outbreaks of human coronavirus (HCoV) at the beginning of the 21^st^ century, indicated coronavirus as a major public health problem worldwide (1, 2). Less than a decade after the last human disease outbreak, caused by the Middle East Respiratory Syndrome Coronavirus (MERS-CoV) in 2012, a new outbreak of the severe acute respiratory syndrome, caused by coronavirus 2 (SARS-CoV-2), is spreading around the world (2). This pandemic is now defined as the 2019 novel coronavirus (COVID-19). The virus was primarily spread by COVID-19 infected individuals. Therefore, the main way to control the spread of COVID-19 disease is to isolate the source of infection. In this regard, the differential screening of COVID-19 highlights the necessity for readily available, accurate and fast screening testing methods (3).

The current gold standard for etiological diagnosis of COVID-19 infection is real time reverse transcription polymerase chain reaction (RT-PCR) in respiratory specimens (4). However, some studies have shown some problems in using this method to detect COVID-19 infection, such as low sensitivity and specificity (5). These studies suggested that causes of these problems may include performing screening outside the diagnostic window, virus mutation and recombination, using insufficiently validated tests, and instrument failure (5). Therefore, more and more studies are taking cross-disciplinary methods to better understand screening and diagnosis of COVID-19 infection in order to improve diagnostic accuracy, for example, by combining clinical evidence, and COVID-19 antibody test results, and interpreting RT-PCR results on the basis of epidemiological and clinical evidence (6). However, none of the current meta-analysis have systematically summarized these strategies (7–9). Although the association between antibodies and SARS-CoV-2 infection has been discussed in some recently published meta-analyses, the studies included in these studies are relatively limited, ranging from 14 to 29 (10–12).

This study aims to (1) systematically summarize the sensitivity and specificity of the major screening methods for the COVID-19, (2) analyze the possible causes of false negatives or false positives as regards the efficacy of the screening methods, and (3) explore how to further improve the sensitivity and specificity of the screening methods. The study will also discuss epidemiological methods that could be used to further help identify more patients with latent infections by investigating their exposure history. It was speculated that, in a public health emergency, a combination of multidisciplinary approaches may be of some help in the control and prevention of COVID-19.

## Methods

### Search Strategy and Selection Criteria

Standard procedures for meta-analyses according to the Cochrane Handbook; and the Preferred Reporting Items for Systematic Reviews and Meta-Analyses (PRISMA) guidelines were used to conduct this study (13). Thus, using these procedures, two independent reviewers (XP and AK) systematically searched in the electronic databases, Web of Science, Cochrane Library, Embase, PubMed, CNKI, and Wangfang, for relevant studies published in May 1^st^, 2022, or earlier. Then, from the search outcomes, they selected eligible studies according to the purpose of this study, using predefined selection criteria. Noteworthy, China has accumulated valuable experience in screening and diagnosing COVID-19, but very much literature related to China on this subject has been published in Chinese language. Therefore, to avoid publication bias, gray literature and studies published in Chinese were included in this study. That is, studies published in English or Chinese were included in this study. In order to retrieve as much literature as possible, the search strategy, among other important terms, included the professional name, COVID-19, and its variant names. Based on both historical and current COVID-19 names, Boolean operators, truncation and wildcards were used appropriately to include all other variant names for COVID-19. The complete search strategy is shown in the Supplementary Materials (Appendix 1). Experienced librarians designed the search strategy and adjusted it to meet the requirements of each of the databases specified above. For example, a search strategy in the database of PubMed was structured as follows using keywords (search terms): (((“COVID-19”[Title/Abstract] OR “2019 novel coronavirus infection”[Title/Abstract] OR “COVID19”[Title/Abstract] OR “coronavirus disease 2019”[Title/Abstract] OR “coronavirus disease-19”[Title/Abstract] OR “2019-nCoV disease”[Title/Abstract] OR “2019 novel coronavirus disease”[Title/Abstract] OR “2019-nCoV infection”[Title/Abstract] OR “coronavirus disease 2019 virus”[Title/Abstract] OR “SARS-CoV-2”[Title/Abstract] OR “SARS2”[Title/Abstract] OR “2019-nCoV”[Title/Abstract] OR “2019 novel coronavirus”[Title/Abstract] OR “severe acute respiratory syndrome coronavirus 2”[Title/Abstract] OR “COVID Asymptomatic Infections”[Title/Abstract]) AND (“Nucleic Acid Detection”[Title/Abstract] OR “Nucleic Acid Probes”[Title/Abstract] OR “Molecular Probes”[Title/Abstract] OR “Nucleic Acid Probes”[Title/Abstract] OR “Reagent Kits, Diagnostic”[Title/Abstract] OR “Reagent Strips”[Title/Abstract] OR “polymerase chain reaction”[Title/Abstract] OR “PCR^*^”[Title/Abstract])) AND (“Serology”[Title/Abstract] OR “Antibody”[Title/Abstract])).

### Study Selection

Selection of studies for this meta-analysis was based on the following inclusion criteria: (1) diagnostic and screening studies; (2) studies reported methods for diagnosing COVID-19; and (3) original studies. Studies were excluded based on the following criteria: (1) conference papers, case reports, letters, or reviews; and (2) studies not on humans.

### Data Extraction

Two reviewers (SW and AK) translated the Chinese articles into English, and entered all the data and removed duplicates in EndNote (version x9.1), Then used custom data extraction tool, EpiData (version 3.0) grids, and extracted (14, 15). The key characteristics of interest were extracted from each eligible study in the EpiData (version 3.0) grids, including first author, year of publication, study area, number of subjects, sensitivity, and specificity. Any differences between the two reviewers were resolved by consensus involving the third reviewer (AL).

### Quality Evaluation

The Quality Assessment of Diagnostic Accuracy Studies-2 checklist (QUADAS-2) was used to assess the quality of the eligible studies. The selected studies were grouped based on their score into high (6–7 points), moderate (4–5 points), and low (0–3 points) quality categories (16, 17).

### Statistical Analysis

In this study, meta-analyses were carried out using MetaDiSc (version 1.4) and R software (version R i386 3.4.2). For the diagnostic meta-analysis, the number of subjects with a true-positive (TP), false-positive (FP), true-negative (TN), and false-negative (FN) values for each study unit was extracted to calculate the pooled sensitivity [TP/(TP+FN)], specificity [TN/(TN+FP)], positive likelihood ratio (PLR) [(sensitivity/(1–sensitivity)], negative likelihood ratio (NLR), [(1–specificity)/specificity)], diagnostic odds ratio (dOR) [PLR/NLR], and their corresponding 95% confidence intervals (CIs) using a bivariate random-effect meta-analysis model (18). The summary receiver operator characteristic (SROC) curve was plotted, and the area under the SROC curve (AUC) was calculated to evaluate the pooled diagnostic performance of IgG and IgM for predicting COVID-19 diagnosis (19, 20). Heterogeneity between enrolled studies was quantified by the *I*^2^ statistic and assessed by the Cochran's Q-statistic. *I*^2^ = 0% indicated no heterogeneity, and *I*^2^ = 100% indicated maximal heterogeneity (21, 22). Publication bias was assessed using the Deeks' funnel plot asymmetry test, when the number of studies reporting meta-analysis results was 10 or more. Finally, in all analyses, the level of significance for the effect size estimation was set at 5%, and all tests were two-sided.

## Results

### Characteristics of Eligible Studies

**Figure 1:**
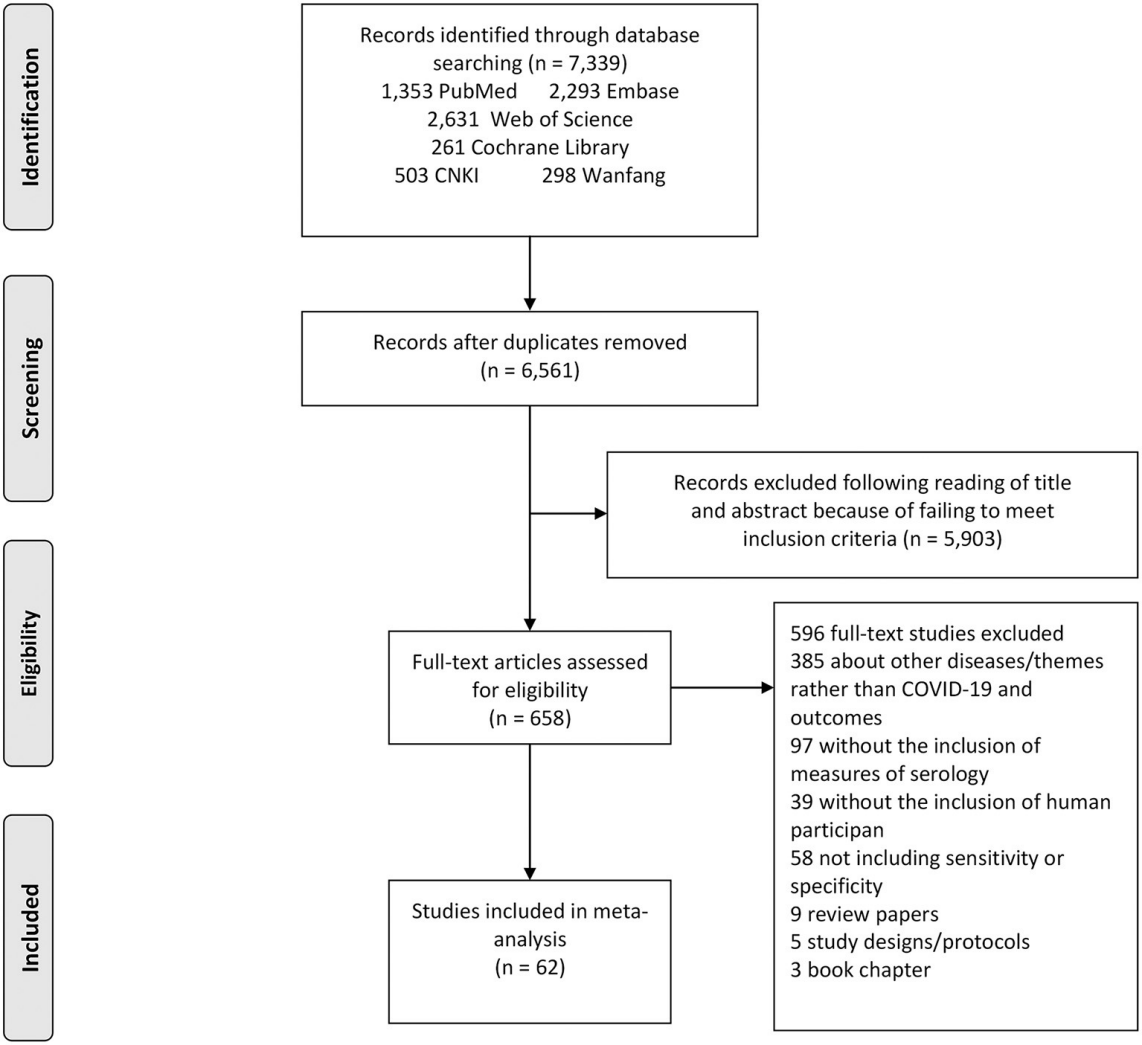
Study selection flow chart. A Preferred Reporting Items for Systematic Reviews and Meta-Analyses (PRISMA) flow chart demonstrating the selection process of articles included in the analysis as well as in the qualitative summary.

The search strategy retrieved a total of 7,339 studies, of which 2,631 from Web of Science, 261 from Cochrane library, 2,293 from Embase, 1,353 were from PubMed, 503 from CNKI and 298 from Wanfang. Following a full review of 658 of these studies, 62 met the inclusion criteria for this meta-analysis. The PRISMA flowchart in Figure 1 shows the number of studies and the selection process.

### Characteristics of Eligible Studies

Sixty-two studies were included with 35,775 participants in our meta-analysis. Characteristics of the eligible studies are shown in Appendix 2. Further, focusing on test method, 62 studies reported on antibody tests. The QUADAS-2 score of these studies varied between 3 and 6, with 27 studies of high quality and 35 of moderate quality. There are 44 studies from Asia, 12 from Europe, 4 from the Americas and 2 from Africa.

### Main Outcomes

**Figure 2:**
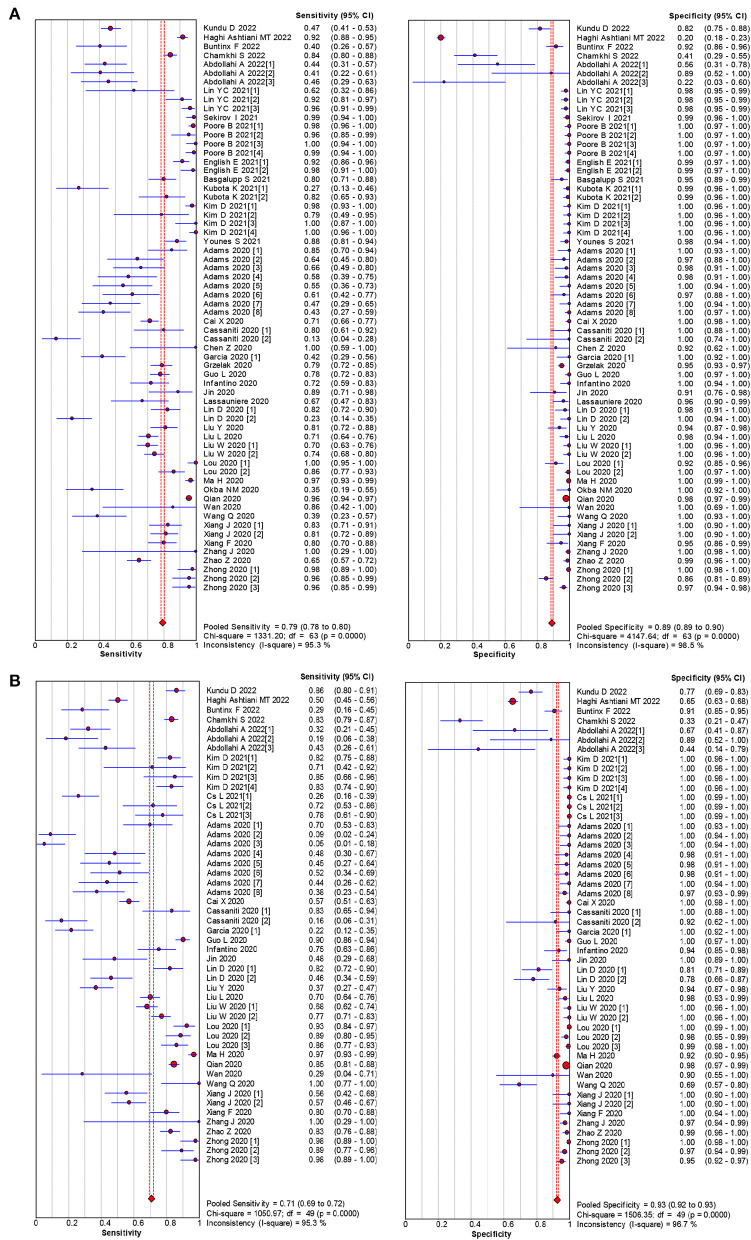
Forest plot of sensitivities and specificities IgG **(A)** and IgM **(B)** for predicting COVID-19 diagnosis.

**Figure 3:**
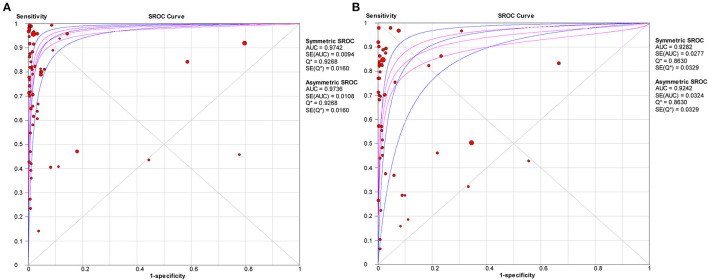
SROC curve with pooled estimates of sensitivity, specificity, and the AUC for all included studies IgG **(A)** and IgM **(B)** for predicting COVID-19 diagnosis. AUC, area under the SROC curve; SROC, summary receiver operator characteristic.

**Figure 4:**
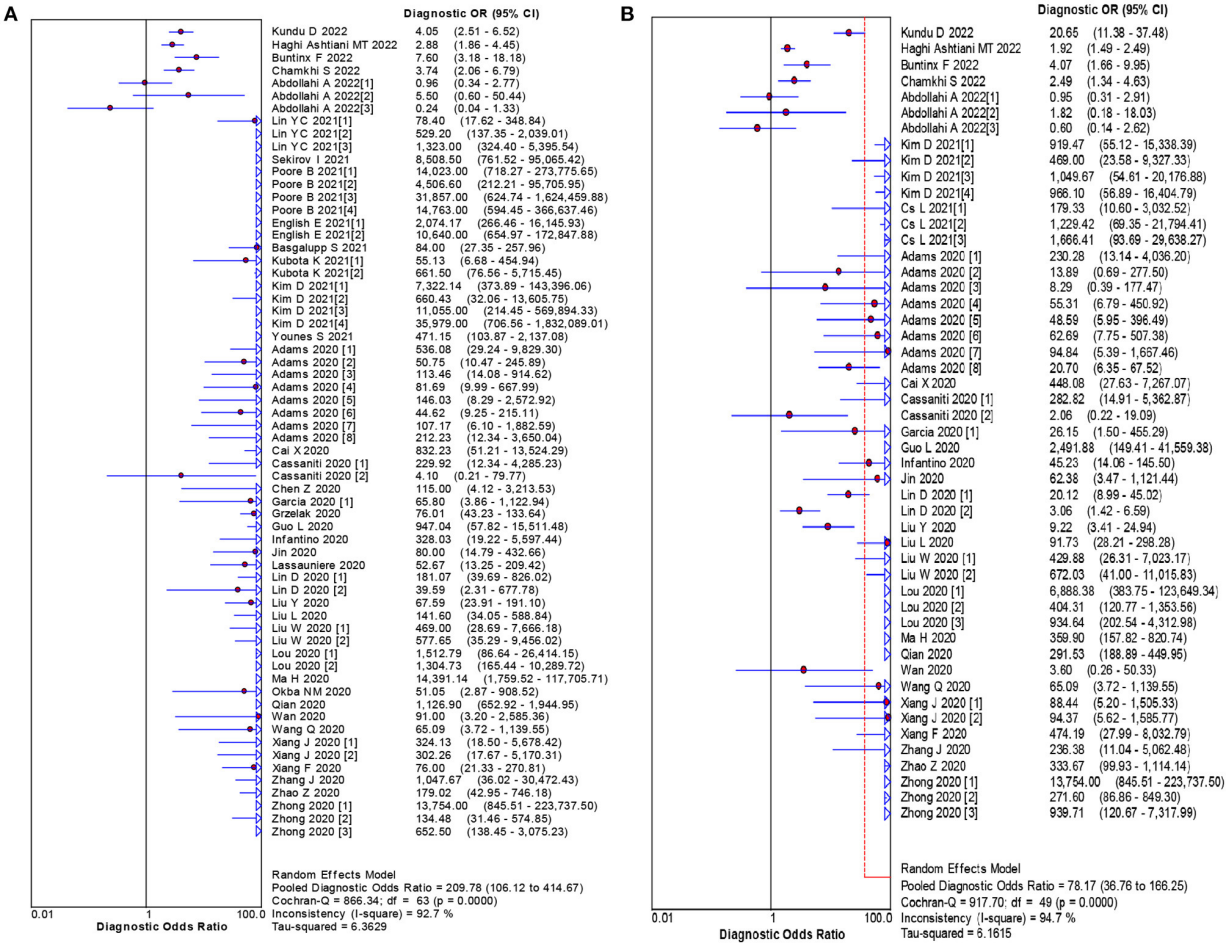
Forest plot of the pooled dOR of IgG **(A)** and IgM **(B)** for predicting COVID-19 diagnosis.

The pooled estimates for sensitivity and specificity of IgG to predicting COVID-19 diagnosis were 0.79 (95% CI: 0.78–0.80) and 0.89 (95% CI: 0.89–0.90), respectively, (Figure 2), corresponding to a PLR of 37.47 (95% CI: 14.59–96.26) (Appendix 3) and an NLR of 0.20 (95% CI: 0.16–0.27) (Appendix 4). The overall AUC was 0.97 (Figure 3) and the dOR was 209.78 (95% CI: 106.12–414.67) (Figure 4).

The pooled estimates for sensitivity and specificity of IgM to predicting COVID-19 diagnosis were 0.71 (95% CI: 0.69–0.72) and 0.93 (95% CI: 0.92–0.93), respectively, (Figure 2), corresponding to a PLR of 20.40 (95% CI: 11.71–35.55) (Appendix 5) and an NLR of 0.33 (95% CI: 0.25–0.42) (Appendix 6). The overall AUC was AUC was 0.93 (Figure 3) and the dOR was 78.17 (95% CI: 36.76–166.25) (Figure 4).

**Figure 5:**
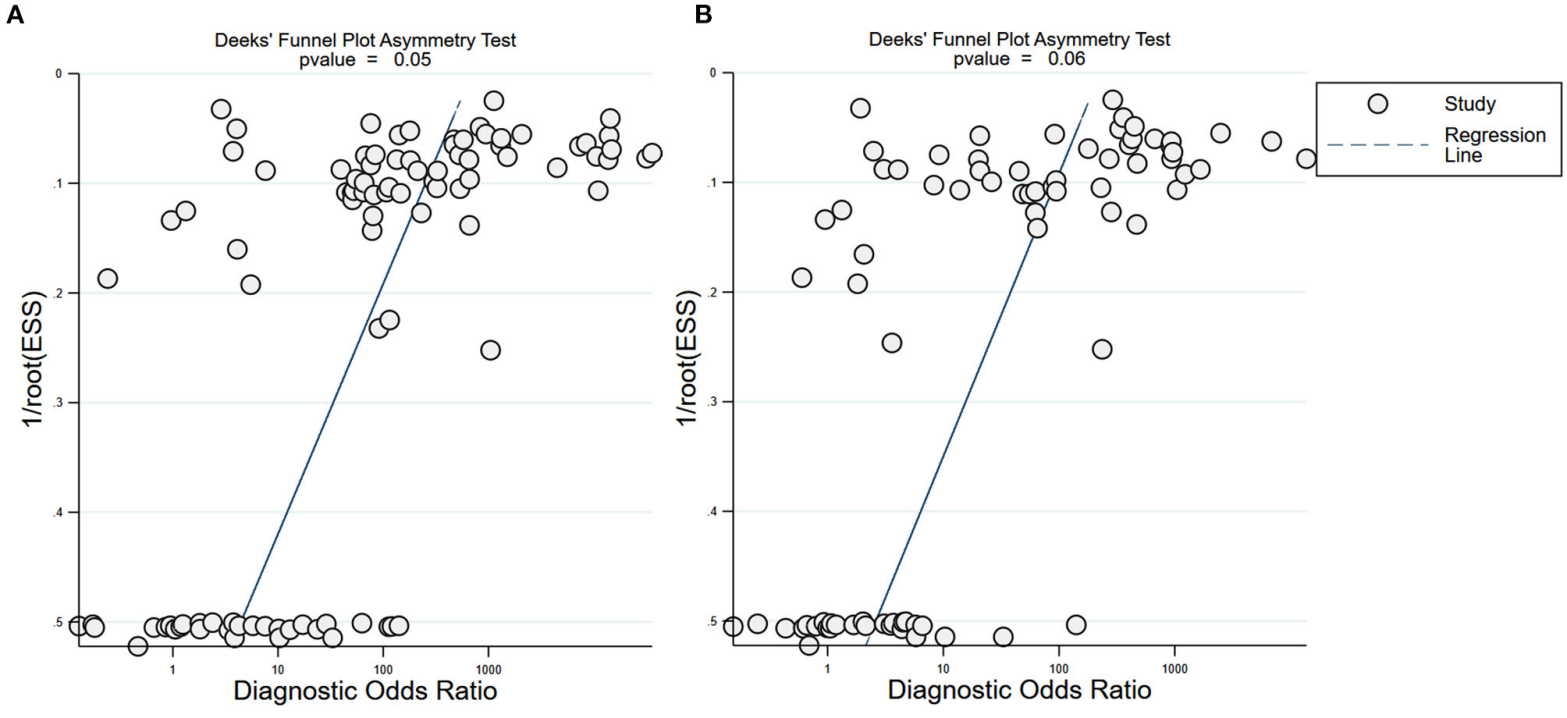
Deeks' funnel plot for IgG and IgM. Deeks' funnel plot to assess publication bias for IgG **(A)** and IgM **(B)**. Plots show study size as a function of effect size for studies included in the meta-analysis. The dots represent each study.

Subgroup analysis showed that there were differences among different regions. The studies in Asia with higher sensitivity of IgG and IgM to predicting COVID-19 diagnosis were 0.80 (95% CI: 0.79–0.81) and 0.73 (95% CI: 0.72–0.75), respectively (Table 1). However, the studies in Europe with lower sensitivity of IgG and IgM to predicting COVID-19 diagnosis were 0.67 (95% CI: 0.64–0.71) and 0.41 (95% CI: 0.37–0.46), respectively (Table 1). The studies in Europe with higher specificity of IgG and IgM to predicting COVID-19 diagnosis were 0.98 (95% CI: 0.97–0.98) and 0.97 (95% CI: 0.96–0.98), respectively, (Table 2). However, the studies in Africa with lower specificity of IgG and IgM to predicting COVID-19 diagnosis were 0.42 (95% CI: 0.29–0.55) and 0.34 (95% CI: 0.22–0.48), respectively, (Table 2). The Deeks' funnel plot for IgG and IgM was symmetrical (Figure 5), and the Deeks' test did not reject the hypothesis that there was no publication bias for IgG (*t* = −1.97, *p* = 0.052) and IgM (*t* = −1.88, *p* = 0.064).

**Table 1:** Subgroup analysis of sensitivities for IgG and IgM to predicting COVID-19 diagnosis.

	**Sub**	**Pooled sensitivity**	**95%Cl**		**Chi-square**	***I^**2**^***
Ig G	All	0.79	0.78	0.80	1331.20	95.30%
Ig G	Asia	0.80	0.79	0.81	905.20	95.70%
Ig G	Europe	0.67	0.64	0.71	213.84	92.10%
Ig G	Africa	0.66	0.62	0.70	178.28	98.30%
Ig G	America	0.95	0.92	0.97	43.07	90.70%
Ig M	All	0.71	0.69	0.72	1050.97	95.30%
Ig M	Asia	0.73	0.72	0.75	688.02	94.90%
Ig M	Europe	0.41	0.37	0.46	134.98	91.10%
Ig M	Africa	0.61	0.57	0.65	170.16	98.20%
Ig M	America	0.79	0.74	0.83	56.94	94.70%

**Table 2:** Subgroup analysis of specificities for IgG and IgM to predicting COVID-19 diagnosis.

	**Sub**	**Pooled specificity**	**95%Cl**		**Chi-square**	***I^**2**^***
Ig G	All	0.89	0.89	0.90	4,147.64	98.50%
Ig G	Asia	0.87	0.86	0.87	3,657.43	98.90%
Ig G	Europe	0.98	0.97	0.98	54.79	69%
Ig G	Africa	0.42	0.29	0.55	0.09	0%
Ig G	America	0.99	0.98	1.00	15.57	74.30%
Ig M	All	0.93	0.92	0.93	1,506.35	96.70%
Ig M	Asia	0.92	0.92	0.93	1,312.61	97.30%
Ig M	Europe	0.97	0.96	0.98	33.29	64%
Ig M	Africa	0.34	0.22	0.48	0.33	0%
Ig M	America	0.50	0.07	0.93	0.00	0%

## Discussion

Although the association between antibodies and SARS-CoV-2 infection has been discussed in some recently published meta-analyses, the studies included in these studies are relatively limited, ranging from 14 to 29 (10–12). This is the maximum sample size, comprehensive, and updated review of the latest advances in the diagnosis of COVID-19 (7, 9, 23). Sixty-two studies were included with 35,775 participants in our meta-analysis. In view of the relatively strong infectivity of COVID-19, early detection, reporting, isolation and treatment are of great significance for the prevention and control of the spread of the infection. Following their encouraging accuracy as shown in the reviewed studies, RT-PCR nucleic acid and serum-specific antibody detection are recommended as the main screening criteria (24). At the same time, different methods of cross-test verification may further improve the sensitivity and specificity, such as serum-specific antibody detection, hence this may further reduce false negatives and false positives, and increase the accuracy of screening.

Furthermore, laboratory confirmed positive cases should meet one of the following two conditions. First, the two targets of COVID-19 (ORF1ab, N or E) in the same specimen should all be positive in real-time fluorescence RT-PCR (25). For example, if one target tests positive, it is necessary to re-sample and re-test it. If it still tests positive after re-sampling, then it is positive. Second, both samples should be positive for a single target by the real-time fluorescent RT-PCR, or both samples of the same type should be positive for a single target in 2 sampling tests, which could be judged as positive. In addition, the results of the reviewed studies suggest that N gene or E gene test could be used as a screening tool in the routine workflow, whereas RdRp gene (ORF1ab) could be used as a diagnostic tool, or it could be directly detected separately (26).

Current studies have shown that nucleic acid detection technology has the characteristics of early diagnosis, high sensitivity and specificity (27). However, in practice, this technology often yields false negatives. Therefore, the potential methods to reduce false negative results from the aspects of sample collection time, sample collection site and nucleic acid extraction process are worth discussing (7). First, mutations in the primers design area of the viral RNA may result in false negative test results. Thus, based on the preceding possibility, we recommend the following: collecting nasopharyngeal swabs within 3–7 days of onset (28), testing sputum samples in patients with negative RT-PCR results from pharyngeal swabs, and suspect or confirm a high probability of infection (29). However, most patients have been sick for more than 1 week at the time of treatment. In this regard, we recommend combining these with serological antibody detection (24, 30).

**Figure 6:**
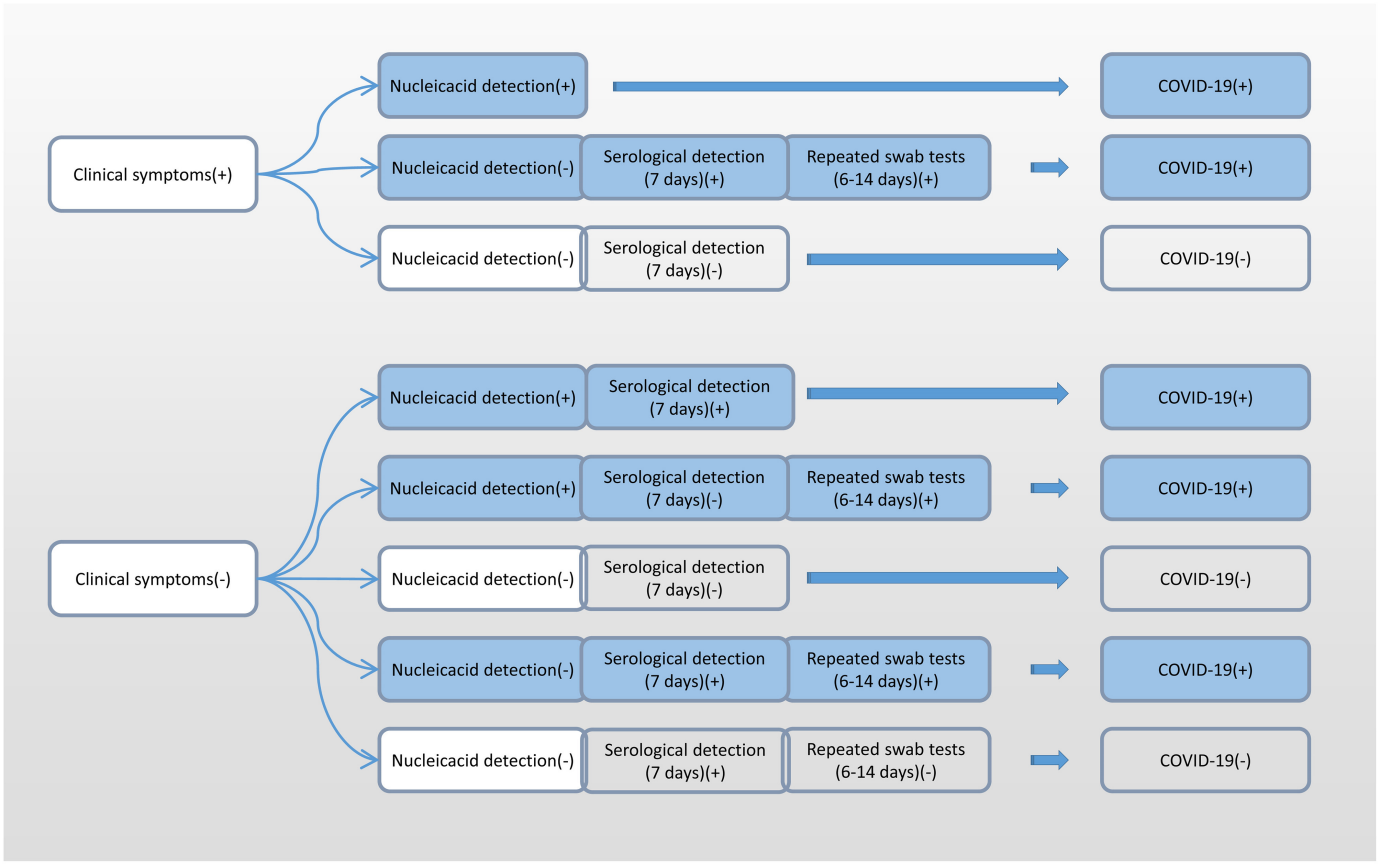
Flow chart of the selection of different detection methods for SARS-CoV-2 at different infection periods. SARS-CoV-2, severe acute respiratory syndrome coronavirus 2.

Additionally, due to the differences in the incubation period of individuals, we recommend multiple nucleic acid tests for clinically symptomatic patients in order to improve the detection rate (31). Alveolar lavage fluid sampling is not recommended because of the risk of trauma and cross-infection. According to the accurate selection of appropriate detection methods at different infection periods, we recommend nucleic acid detection immediately after showing clinical symptoms, and serological detection 7 days after having shown clinical symptoms (Figure 6).

**Figure 7:**
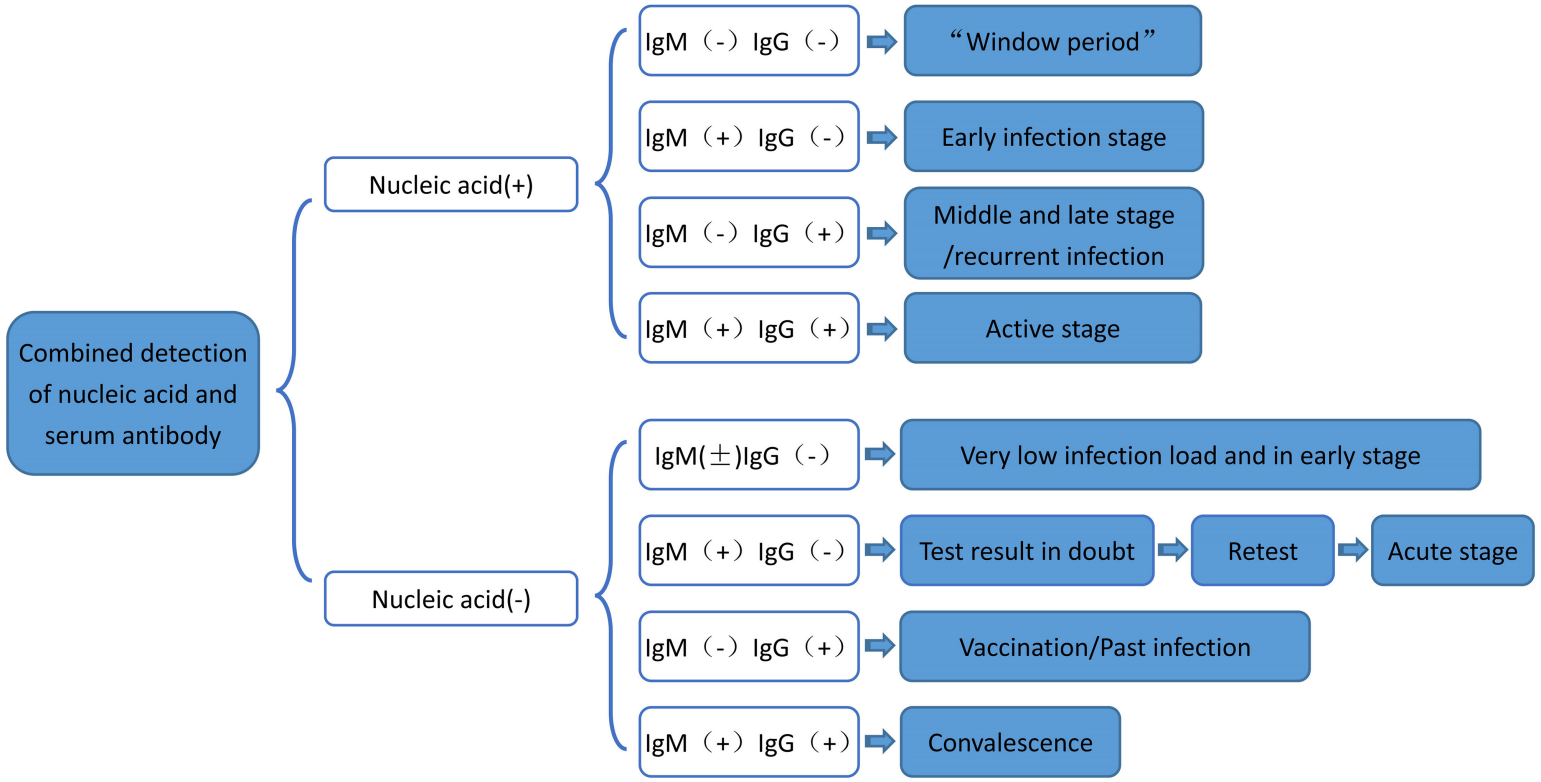
Flow chart of serum-specific antibody binding RT-PCR nucleic acid detection. RT-PCR, Real-time fluorescence quantification reverse transcriptase; COVID-19, 2019 novel coronavirus.

Moreover, studies have shown that both IgG and IgM positive rates increase after COVID-19 infection (24). Serum specific antibody can be used as an important indicator to evaluate COVID-19 infection, and IgM can effectively diagnose and exclude COVID-19 nucleic acid negative patients (24). Therefore, dynamic monitoring of viral IgM or IgG can be considered as a complementary means for nucleic acid detection (32). The flow chart of serum-specific antibody binding RT-PCR nucleic acid detection is summarized in Figure 7. Studies have shown that if nucleic acid is positive, then IgG and IgM are all negative, while if nucleic acid and IgM are positive, but IgG is negative, then patients are in the early stage of infection (32). In addition, patients with nucleic acid and IgG positive, but IgM negative, may be in the middle or late stage of COVID-19 infection or recurrent infection. Also, patients with nucleic acid positive, IgM positive, and IgG positive may be in the active period of the infection (33). Further, nucleic acid negative, IgM positive, and IgG negative results most likely suggest acute stage of the COVID-19 infection (34). Besides, nucleic acid negative, IgM negative, and IgG positive suggest that the patient may have been previously infected with COVID-19 (24).

Subgroup analysis showed that there were differences among different regions. The studies in Asia with higher sensitivity of IgG and IgM to predicting COVID-19 diagnosis. However, the studies in Europe with lower sensitivity of IgG and IgM to predicting COVID-19 diagnosis. The studies in Europe with higher specificity of IgG and IgM to predicting COVID-19 diagnosis. However, the studies in Africa with lower specificity of IgG and IgM to predicting COVID-19 diagnosis. These results may be related to the intensity of immune response between different races to produce antibodies against the SARS-CoV-2 infection (35, 36). Previous study has shown that Hispanics and Blacks had higher rates of SARS-CoV-2 antibodies than Whites, indicating that SARS-CoV-2 spread disproportionately in racial and ethnic minorities during the COVID-19 pandemic (37, 38). Future studies should further explore the possible molecular mechanisms (39, 40).

The biggest advantage of the serum antibody kit is that the sample collection is easy, the sample processing and the experimental operation is simple, the test time is short, hence the speed is fast, and it is suitable for large-sample screening. However, antibodies also have the limitation of false negatives (33, 34), particularly resulting from (1) the type of preservatives and anticoagulants used; (2) improper specimen preservation; (3) laboratory operator error or improper preservation of the kit; and (4) too fast chromatography. In relation to (1), treating specimens with preservative sodium azide and anticoagulant EDTA can inhibit the activity of horseradish peroxidase (HRP), resulting in false negative enzyme linked immunosorbent assay (ELISA). As regards (2), when the specimen is kept in the refrigerator for too long, the IgG in the serum polymerizes into a polymer, and the immune activity of the antibody is weakened. Considering (3), if the reagent is not balanced to room temperature before use, the reagent dosage is insufficient, and the incubation time or temperature is insufficient; then the reagent is not stored as required, as a result it becomes contaminated or ineffective. Finally, taking (4), if the antigen antibody complex has not yet bound with the antibody on the detection line, then it is more likely that this occurs out of the detection line, resulting in false negatives. In practice, these problems should be avoided so that false negative results should be minimized.

## Conclusions

Multidisciplinary cooperation can improve the diagnostic efficiency of COVID-19. We recommend the use of RT-PCR nucleic acid and serum-specific antibody detection as the main screening criteria. Through standard sampling, sample processing, combined accurate selection of appropriate detection methods in different infection stages of laboratory methods, sensitivity of detection can be improved.

## Data Availability Statement

The original contributions presented in the study are included in the article/Supplementary Material, further inquiries can be directed to the corresponding authors.

## Author Contributions

XP, PJ, and, AL contributed to the study design, while YF and AK contributed to the data collection. Interpretation of results was performed by AL and HL, whereas XP, YC, AL, and SW drafted the manuscript and edited the language. All authors participated in the critical revisions and approved the final version of the manuscript.

## Funding

This study was supported by the International Institute of Spatial Lifecourse Health (ISLE), Wuhan University Specific Fund for Major School-level Internationalization Initiatives (WHU-GJZDZX-PT07), Hunan Provincial Key Laboratory of Clinical Epidemiology, and the Hunan Provincial Key Research and Development Program (2018SK2065), China.

## Conflict of Interest

The authors declare that the research was conducted in the absence of any commercial or financial relationships that could be construed as a potential conflict of interest.

## Publisher's Note

All claims expressed in this article are solely those of the authors and do not necessarily represent those of their affiliated organizations, or those of the publisher, the editors and the reviewers. Any product that may be evaluated in this article, or claim that may be made by its manufacturer, is not guaranteed or endorsed by the publisher.
