# Supplementary material for: Auxiliary Screening COVID-19 by Serology
Source: Front Public Health. 2022 Aug 2;10:819841. doi: 10.3389/fpubh.2022.819841 (PMC9380738; doi:10.3389/fpubh.2022.819841)

**Supplementary appendix**

**Contents**

**Appendix 1. Search strategy and search results**

**Appendix 2. Characteristics and data of included studies for antibody tests**

**Appendix 3. Forest plot of likelihood ratios for positive test results of Ig G for predicting COVID-19 diagnosis**

**Appendix 4. Forest plot of likelihood ratios for negative test results of Ig G for predicting COVID-19 diagnosis**

**Appendix 5. Forest plot of likelihood ratios for positive test results of Ig M for predicting COVID-19 diagnosis**

**Appendix 6. Forest plot of likelihood ratios for negative test results of Ig M for predicting COVID-19 diagnosis**

**Appendix 1. Search strategy and search results**

The search strategy includes all possible combinations of keywords in the title/abstract from the following two groups:

(#1)“COVID-19” or “2019 novel coronavirus infection” or “COVID19” or “coronavirus disease 2019” or “coronavirus disease-19” or “2019-nCoV disease” or “2019 novel coronavirus disease” or “2019-nCoV infection” or “coronavirus disease 2019 virus” or “SARS-CoV-2” or “SARS2” or “2019-nCoV” or “2019 novel coronavirus” or “severe acute respiratory syndrome coronavirus 2” or “COVID Asymptomatic Infections”

(#2) “Nucleic Acid Detection” or “Nucleic Acid Probes” or “Nucleic Acid Probes” or “Reagent Kits, Diagnostic” or “Reagent Strips” or “polymerase chain reaction” or “PCR*”

(#3) “Serology” or “Antibody”

(#1 AND #2 AND #3)

Chinese database

(#1) “COVID-19”或“2019新型冠状病毒”或“COVID 19”或“2019冠状病毒”或“冠状病毒病-19”或“2019 nCoV病”或 “2019 nCoV感染”或 “2019冠状病毒病病毒”或“SARS-CoV-2”或“SARS2”或“2019 nCoV”或“2019新型冠状病毒”或“严重急性呼吸综合征冠状病毒2” 或“COVID 19, 无症状感染者” 或“COVID 19, 隐匿感染者” 或“COVID 19, 隐藏感染者” 或“COVID 19, 隐性感染者”)

(#2) “核酸检测”或“核酸探针”或“分子探针”或“诊断试剂盒”或“聚合酶链式反应”或“PCR”

(#3) “血清” or “抗体”

(#1 AND #2 AND #3)

**The search results as following:**

Cochrane library

| #1 | “COVID 19” or “2019 novel corona virus infection” or “COVID19” or “corona virus disease 2019” or “corona virus disease 19” or “2019 nCoV disease” or “2019 novel corona virus disease” or “2019 nCoV infection” or “corona virus disease 2019 virus” or “SARS CoV 2” or “SARS2” or “2019 nCoV” or “2019 novel corona virus” or “severe acute respiratory syndrome corona virus 2” or “COVID, Asymptomatic Infections” |
| --- | --- |
| #2 | “Nucleic Acid Detection” or “Nucleic Acid Probes” or “Molecular Probes” or “Nucleic Acid Probes” or “Reagent Kits, Diagnostic” or “Reagent Strips” or “polymerase chain reaction” or “PCR*” |
| #3 | “Serology” or “Antibody” |
|  | #1 AND #2 AND #3 |

Web of science

| #1 | TS= (“COVID 19” or “2019 novel corona virus infection” or “COVID19” or “corona virus disease 2019” or “corona virus disease 19” or “2019 nCoV disease” or “2019 novel corona virus disease” or “2019 nCoV infection” or “corona virus disease 2019 virus” or “SARS CoV 2” or “SARS2” or “2019 nCoV” or “2019 novel corona virus” or “severe acute respiratory syndrome corona virus 2” or “COVID, Asymptomatic Infections”) |
| --- | --- |
| #2 | TS= (“Nucleic Acid Detection” or “Nucleic Acid Probes” or “Molecular Probes” or “Nucleic Acid Probes” or “Reagent Kits, Diagnostic” or “Reagent Strips” or “polymerase chain reaction” or “PCR*” |
| #3 | TS= (“Serology” or “Antibody” ) |
|  | #1 AND #2 AND #3 |
|  | “COVID 19” or “2019 novel corona virus infection” or “COVID19” or “corona virus disease 2019” or “corona virus disease 19” or “2019 nCoV disease” or “2019 novel corona virus disease” or “2019 nCoV infection” or “corona virus disease 2019 virus” or “SARS CoV 2” or “sarsr” or “2019 nCoV” or “2019 novel corona virus” or “severe acute respiratory syndrome corona virus 2” or “COVID, Asymptomatic Infections” (Topic) and “Nucleic Acid Detection” or “Nucleic Acid Probes” or “Molecular Probes” or “Nucleic Acid Probes” or “Reagent Kits, Diagnostic” or “Reagent Strips” or “polymerase chain reaction” or “PCR*” (Topic) and “Serology” or “Antibody” (Topic) |

**Pubmed**

| #1 | “COVID-19”[Title/Abstract] OR “2019 novel coronavirus infection”[Title/Abstract] OR “COVID19”[Title/Abstract] OR “coronavirus disease 2019”[Title/Abstract] OR “coronavirus disease-19”[Title/Abstract] OR “2019-nCoV disease”[Title/Abstract] OR “2019 novel coronavirus disease”[Title/Abstract] OR “2019-nCoV infection”[Title/Abstract] OR “coronavirus disease 2019 virus”[Title/Abstract] OR “SARS-CoV-2”[Title/Abstract] OR “SARS2”[Title/Abstract] OR “2019-nCoV”[Title/Abstract] OR “2019 novel coronavirus”[Title/Abstract] OR “severe acute respiratory syndrome coronavirus 2”[Title/Abstract] OR “COVID Asymptomatic Infections”[Title/Abstract] |
| --- | --- |
| #2 | “Nucleic Acid Detection”[Title/Abstract] OR “Nucleic Acid Probes”[Title/Abstract] OR “Molecular Probes”[Title/Abstract] OR “Nucleic Acid Probes”[Title/Abstract] OR “Reagent Kits, Diagnostic”[Title/Abstract] OR “Reagent Strips”[Title/Abstract] OR “polymerase chain reaction”[Title/Abstract] OR “PCR*”[Title/Abstract] |
| #3 | “Serology”[Title/Abstract] OR “Antibody”[Title/Abstract] |
|  | #1 AND #2 AND #3 |
|  | ((("COVID-19"[Title/Abstract] OR "2019 novel coronavirus infection"[Title/Abstract] OR "COVID19"[Title/Abstract] OR "coronavirus disease 2019"[Title/Abstract] OR "coronavirus disease-19"[Title/Abstract] OR "2019-nCoV disease"[Title/Abstract] OR "2019 novel coronavirus disease"[Title/Abstract] OR "2019-nCoV infection"[Title/Abstract] OR "coronavirus disease 2019 virus"[Title/Abstract] OR "SARS-CoV-2"[Title/Abstract] OR "SARS2"[Title/Abstract] OR "2019-nCoV"[Title/Abstract] OR "2019 novel coronavirus"[Title/Abstract] OR "severe acute respiratory syndrome coronavirus 2"[Title/Abstract] OR "COVID Asymptomatic Infections"[Title/Abstract]) AND ("Nucleic Acid Detection"[Title/Abstract] OR "Nucleic Acid Probes"[Title/Abstract] OR "Molecular Probes"[Title/Abstract] OR "Nucleic Acid Probes"[Title/Abstract] OR "Reagent Kits, Diagnostic"[Title/Abstract] OR "Reagent Strips"[Title/Abstract] OR "polymerase chain reaction"[Title/Abstract] OR "PCR*"[Title/Abstract])) AND ("Serology"[Title/Abstract] OR "Antibody"[Title/Abstract])) |

**Embase**

| #1 | “COVID 19” or “2019 novel corona virus infection” or “COVID19” or “corona virus disease 2019” or “corona virus disease 19” or “2019 nCoV disease” or “2019 novel corona virus disease” or “2019 nCoV infection” or “corona virus disease 2019 virus” or “SARS CoV 2” or “SARS2” or “2019 nCoV” or “2019 novel corona virus” or “severe acute respiratory syndrome corona virus 2” or “COVID, Asymptomatic Infections” |
| --- | --- |
| #2 | “Nucleic Acid Detection” or “Nucleic Acid Probes” or “Acid Probes Nucleic” or “Probes Nucleic Acid” or “Molecular Probes” or “Nucleic Acid Probes” or “Reagent Kits, Diagnostic” or “Reagent Strips” or “polymerase chain reaction” or “PCR” |
| #3 | “Serology” or “Antibody” |
|  | #1 AND #2 AND #3 |
|  | (“COVID 19” or “2019 novel corona virus infection” or “COVID19” or “corona virus disease 2019” or “corona virus disease 19” or “2019 nCoV disease” or “2019 novel corona virus disease” or “2019 nCoV infection” or “corona virus disease 2019 virus” or “SARS CoV 2” or “SARS2” or “2019 nCoV” or “2019 novel corona virus” or “severe acute respiratory syndrome corona virus 2” or “COVID, Asymptomatic Infections”):ab AND (“Nucleic Acid Detection” or “Nucleic Acid Probes” or “Acid Probes Nucleic” or “Probes Nucleic Acid” or “Molecular Probes” or “Nucleic Acid Probes” or “Reagent Kits, Diagnostic” or “Reagent Strips” or “polymerase chain reaction” or “PCR” ):ab AND (“Serology” or “Antibody”):ab |

CNKI

| #1 | SU =“COVID-19” + “2019新型冠状病毒” + “COVID 19” + “2019冠状病毒” + “冠状病毒病-19” + “2019 nCoV病” + “2019 nCoV感染” + “2019冠状病毒病病毒” + “SARS-CoV-2” + “SARS2” + “2019 nCoV” + “2019新型冠状病毒” + “严重急性呼吸综合征冠状病毒2” + “COVID 19, 无症状感染者” + “COVID 19, 隐匿感染者” + “COVID 19, 隐藏感染者” + “COVID 19, 隐性感染者” |
| --- | --- |
| #2 | SU = "核酸检测" + "核酸探针" +"分子探针" + "诊断试剂盒" + "聚合酶链式反应" + "PCR" |
| #3 | FT =“血清”+ “抗体” |
|  | #1 AND #2 AND #3 |
|  | (SU =“COVID-19” + “2019新型冠状病毒” + “COVID 19” + “2019冠状病毒” + “冠状病毒病-19” + “2019 nCoV病” + “2019 nCoV感染” + “2019冠状病毒病病毒” + “SARS-CoV-2” + “SARS2” + “2019 nCoV” + “2019新型冠状病毒” + “严重急性呼吸综合征冠状病毒2” + “COVID 19, 无症状感染者” + “COVID 19, 隐匿感染者” + “COVID 19, 隐藏感染者” + “COVID 19, 隐性感染者”) AND (SU = "核酸检测" + "核酸探针" +"分子探针" + "诊断试剂盒" + "聚合酶链式反应" + "PCR" ) AND (FT =“血清”+ “抗体”) |

Wanfang

| #1 | 主题: “COVID-19”or“2019新型冠状病毒”or“COVID 19”or“2019冠状病毒”or“冠状病毒病-19”or“2019 nCoV病”or “2019 nCoV感染”or “2019冠状病毒病病毒”or“SARS-CoV-2”or“SARS2”or“2019 nCoV”or“2019新型冠状病毒”or“严重急性呼吸综合征冠状病毒2” or“COVID 19, 无症状感染者” or“COVID 19, 隐匿感染者”or“COVID 19, 隐藏感染者”or“COVID 19, 隐性感染者” |
| --- | --- |
| #2 | 主题: "核酸检测" or "核酸探针" or "分子探针" or "诊断试剂盒" or "聚合酶链式反应" or "PCR" |
| #3 | 主题: “血清” or “抗体” |
|  | #1 AND #2 AND #3 |
|  | (主题: “COVID-19”or“2019新型冠状病毒”or“COVID 19”or“2019冠状病毒”or“冠状病毒病-19”or“2019 nCoV病”or “2019 nCoV感染”or “2019冠状病毒病病毒”or“SARS-CoV-2”or“SARS2”or“2019 nCoV”or“2019新型冠状病毒”or“严重急性呼吸综合征冠状病毒2” or“COVID 19, 无症状感染者” or“COVID 19, 隐匿感染者”or“COVID 19, 隐藏感染者”or“COVID 19, 隐性感染者”) AND (主题: "核酸检测" or "核酸探针" or "分子探针" or "诊断试剂盒" or "聚合酶链式反应" or "PCR") AND (主题: “血清” or “抗体” ) |

**Appendix 2. Characteristics and data of included studies for antibody tests**

| **Ref** | **Study** | **Test Method** | **TP** | **FP** | **FN** | **TN** | **Region** |
| --- | --- | --- | --- | --- | --- | --- | --- |
| **(1)** | Kundu D 2022 | Antibody tests (IgG) | 136 | 27 | 153 | 123 | India |
| **(1)** | Kundu D 2022 | Antibody tests (IgM) | 132 | 35 | 21 | 115 | India |
| **(2)** | Haghi Ashtiani MT 2022 | Antibody tests (IgG) | 283 | 841 | 25 | 214 | Iran |
| **(2)** | Haghi Ashtiani MT 2022 | Antibody tests (IgM) | 155 | 364 | 153 | 691 | Iran |
| **(3)** | Buntinx F 2022 | Antibody tests (IgG) | 17 | 11 | 25 | 123 | Belgium |
| **(3)** | Buntinx F 2022 | Antibody tests (IgM) | 12 | 12 | 30 | 122 | Belgium |
| **(4)** | Chamkhi S 2022 | Antibody tests (IgG) | 297 | 34 | 56 | 24 | Tunisia |
| **(4)** | Chamkhi S 2022 | Antibody tests (IgM) | 284 | 38 | 57 | 19 | Tunisia |
| **(5)** | Abdollahi A 2022[1] | Antibody tests (IgG) | 27 | 8 | 35 | 10 | Iran |
| **(5)** | Abdollahi A 2022[2] | Antibody tests (IgG) | 11 | 1 | 16 | 8 | Iran |
| **(5)** | Abdollahi A 2022[3] | Antibody tests (IgG) | 16 | 7 | 19 | 2 | Iran |
| **(5)** | Abdollahi A 2022[4] | Antibody tests (IgM) | 20 | 6 | 42 | 12 | Iran |
| **(5)** | Abdollahi A 2022[5] | Antibody tests (IgM) | 5 | 1 | 22 | 8 | Iran |
| **(5)** | Abdollahi A 2022[6] | Antibody tests (IgM) | 15 | 5 | 20 | 4 | Iran |
| **(6)** | Ozturk A 2021 | Antibody tests (IgG/IgM) | 59 | 6 | 190 | 65 | Turkey |
| **(7)** | Diani E 2021[1] | Antibody tests (IgG/IgM) | 103 | 0 | 48 | 51 | Italy |
| **(7)** | Diani E 2021[2] | Antibody tests (IgG/IgM) | 86 | 0 | 8 | 51 | Italy |
| **(8)** | Kızıloglu I 2021 | Antibody tests (IgG/IgM) | 41 | 16 | 30 | 94 | Turkey |
| **(9)** | Lin YC 2021[1] | Antibody tests (IgG) | 8 | 4 | 5 | 196 | China |
| **(9)** | Lin YC 2021[2] | Antibody tests (IgG) | 54 | 4 | 5 | 196 | China |
| **(9)** | Lin YC 2021[3] | Antibody tests (IgG) | 108 | 4 | 4 | 196 | China |
| **(10)** | Sekirov I 2021 | Antibody tests (IgG) | 91 | 2 | 1 | 187 | Canada |
| **(11)** | Poore B 2021[1] | Antibody tests (IgG) | 189 | 0 | 3 | 129 | United States |
| **(11)** | Poore B 2021[2] | Antibody tests (IgG) | 43 | 0 | 2 | 129 | United States |
| **(11)** | Poore B 2021[3] | Antibody tests (IgG) | 61 | 0 | 0 | 129 | United States |
| **(11)** | Poore B 2021[4] | Antibody tests (IgG) | 85 | 0 | 1 | 129 | United States |
| **(12)** | English E 2021[1] | Antibody tests (IgG) | 131 | 1 | 12 | 190 | United Kingdom |
| **(12)** | English E 2021[2] | Antibody tests (IgG) | 56 | 1 | 1 | 190 | United Kingdom |
| **(13)** | Basgalupp S 2021 | Antibody tests (IgG) | 76 | 4 | 19 | 84 | Brazil |
| **(14)** | Kubota K 2021[1] | Antibody tests (IgG) | 9 | 1 | 24 | 147 | Japan |
| **(14)** | Kubota K 2021[2] | Antibody tests (IgG) | 27 | 1 | 6 | 147 | Japan |
| **(15)** | Kim D 2021[1] | Antibody tests (IgG) | 127 | 0 | 3 | 100 | korea |
| **(15)** | Kim D 2021[2] | Antibody tests (IgG) | 11 | 0 | 3 | 100 | korea |
| **(15)** | Kim D 2021[3] | Antibody tests (IgG) | 27 | 0 | 0 | 100 | korea |
| **(15)** | Kim D 2021[4] | Antibody tests (IgG) | 89 | 0 | 0 | 100 | korea |
| **(15)** | Kim D 2021[5] | Antibody tests (IgM) | 107 | 0 | 23 | 100 | korea |
| **(15)** | Kim D 2021[6] | Antibody tests (IgM) | 10 | 0 | 4 | 100 | korea |
| **(15)** | Kim D 2021[7] | Antibody tests (IgM) | 23 | 0 | 4 | 100 | korea |
| **(15)** | Kim D 2021[8] | Antibody tests (IgM) | 74 | 0 | 15 | 100 | korea |
| **(16)** | Younes S 2021 | Antibody tests (IgG) | 98 | 2 | 13 | 125 | Qatar |
| **(17)** | Boum Y 2021[1] | Antibody tests (IgG) | 14 | 0 | 83 | 0 | Cameroon |
| **(17)** | Boum Y 2021[2] | Antibody tests (IgG) | 24 | 0 | 28 | 0 | Cameroon |
| **(17)** | Boum Y 2021[3] | Antibody tests (IgG) | 134 | 0 | 74 | 0 | Cameroon |
| **(17)** | Boum Y 2021[4] | Antibody tests (IgM) | 19 | 0 | 78 | 0 | Cameroon |
| **(17)** | Boum Y 2021[5] | Antibody tests (IgM) | 23 | 0 | 29 | 0 | Cameroon |
| **(17)** | Boum Y 2021[6] | Antibody tests (IgM) | 100 | 0 | 108 | 0 | Cameroon |
| **(18)** | Cs L 2021[1] | Antibody tests (IgG) | 7 | 0 | 58 | 0 | Singapore |
| **(18)** | Cs L 2021[2] | Antibody tests (IgG) | 25 | 0 | 7 | 0 | Singapore |
| **(18)** | Cs L 2021[3] | Antibody tests (IgG) | 35 | 0 | 1 | 0 | Singapore |
| **(18)** | Cs L 2021[4] | Antibody tests (IgM) | 17 | 0 | 48 | 248 | Singapore |
| **(18)** | Cs L 2021[5] | Antibody tests (IgM) | 23 | 0 | 9 | 248 | Singapore |
| **(18)** | Cs L 2021[6] | Antibody tests (IgM) | 28 | 0 | 8 | 248 | Singapore |
| **(19)** | Adams 2020 [1] | Antibody tests (IgG) | 34 | 0 | 6 | 50 | UK |
| **(19)** | Adams 2020 [2] | Antibody tests (IgG) | 21 | 2 | 12 | 58 | UK |
| **(19)** | Adams 2020 [3] | Antibody tests (IgG) | 25 | 1 | 13 | 59 | UK |
| **(19)** | Adams 2020 [4] | Antibody tests (IgG) | 18 | 1 | 13 | 59 | UK |
| **(19)** | Adams 2020 [5] | Antibody tests (IgG) | 17 | 0 | 14 | 60 | UK |
| **(19)** | Adams 2020 [6] | Antibody tests (IgG) | 20 | 2 | 13 | 58 | UK |
| **(19)** | Adams 2020 [7] | Antibody tests (IgG) | 15 | 0 | 17 | 60 | UK |
| **(19)** | Adams 2020 [8] | Antibody tests (IgG) | 17 | 0 | 23 | 142 | UK |
| **(20)** | Cai X 2020 | Antibody tests (IgG) | 197 | 0 | 79 | 167 | China |
| **(21)** | Cassaniti 2020 [1] | Antibody tests (IgG) | 24 | 0 | 6 | 30 | Italy |
| **(21)** | Cassaniti 2020 [2] | Antibody tests (IgG) | 5 | 0 | 33 | 12 | Italy |
| **(22)** | Chen Z 2020 | Antibody tests (IgG) | 7 | 1 | 0 | 11 | China |
| **(23)** | Du 2020 | Antibody tests (IgG) | 60 | 0 | 0 | 0 | China |
| **(24)** | Freeman 2020 | Antibody tests (IgG) | 94 | 0 | 5 | 0 | USA |
| **(25)** | Gao Y 2020 | Antibody tests (IgG) | 35 | 0 | 3 | 0 | China |
| **(26)** | Gao HX 2020 [1] | Antibody tests (IgG) | 19 | 0 | 18 | 0 | China |
| **(26)** | Gao HX 2020 [2] | Antibody tests (IgG) | 19 | 0 | 18 | 0 | China |
| **(26)** | Gao HX 2020 [3] | Antibody tests (IgG) | 24 | 0 | 13 | 0 | China |
| **(27)** | Garcia 2020 [1] | Antibody tests (IgG) | 23 | 0 | 32 | 45 | Spain |
| **(27)** | Garcia 2020 [2] | Antibody tests (IgG) | 56 | 0 | 7 | 0 | Spain |
| **(28)** | Grzelak 2020 | Antibody tests (IgG) | 127 | 23 | 34 | 468 | France |
| **(29)** | Guo L 2020 | Antibody tests (IgG) | 162 | 0 | 46 | 135 | China |
| **(30)** | Infantino 2020 | Antibody tests (IgG) | 44 | 0 | 17 | 64 | Italy |
| **(31)** | Jia 2020 | Antibody tests (IgG) | 31 | 0 | 26 | 0 | China |
| **(32)** | Jin 2020 | Antibody tests (IgG) | 24 | 3 | 3 | 30 | China |
| **(33)** | Lassauniere 2020 | Antibody tests (IgG) | 20 | 3 | 10 | 79 | Denmark |
| **(34)** | Li Z 2020 | Antibody tests (IgG) | 280 | 0 | 117 | 0 | China |
| **(35)** | Lin D 2020 [1] | Antibody tests (IgG) | 65 | 2 | 14 | 78 | China |
| **(35)** | Lin D 2020 [2] | Antibody tests (IgG) | 15 | 0 | 50 | 64 | China |
| **(36)** | Lippi 2020 [1] | Antibody tests (IgG) | 19 | 0 | 29 | 0 | Italy |
| **(36)** | Lippi 2020 [2] | Antibody tests (IgG) | 7 | 0 | 41 | 0 | Italy |
| **(37)** | Liu Y 2020 | Antibody tests (IgG) | 77 | 5 | 18 | 79 | China |
| **(38)** | Liu L 2020 | Antibody tests (IgG) | 168 | 2 | 70 | 118 | China |
| **(39)** | Liu R 2020 | Antibody tests (IgG) | 129 | 0 | 4 | 0 | China |
| **(40)** | Liu W 2020 [1] | Antibody tests (IgG) | 150 | 0 | 64 | 100 | China |
| **(40)** | Liu W 2020 [2] | Antibody tests (IgG) | 159 | 0 | 55 | 100 | China |
| **(41)** | Long 2020 | Antibody tests (IgG) | 287 | 0 | 76 | 0 | China |
| **(42)** | Lou 2020 [1] | Antibody tests (IgG) | 71 | 9 | 0 | 100 | China |
| **(42)** | Lou 2020 [2] | Antibody tests (IgG) | 69 | 1 | 11 | 208 | China |
| **(43)** | Ma H 2020 | Antibody tests (IgG) | 209 | 1 | 7 | 482 | China |
| **(44)** | Okba NM 2020 | Antibody tests (IgG) | 11 | 0 | 20 | 45 | France |
| **(45)** | Padoan 2020 | Antibody tests (IgG) | 57 | 0 | 13 | 0 | Italy |
| **(46)** | Pan Y 2020 | Antibody tests (IgG) | 60 | 0 | 48 | 0 | China |
| **(47)** | Qian 2020 | Antibody tests (IgG) | 531 | 30 | 24 | 1528 | China |
| **(48)** | To KK 2020 [1] | Antibody tests (IgG) | 15 | 0 | 1 | 0 | China |
| **(48)** | To KK 2020 [2] | Antibody tests (IgG) | 16 | 0 | 0 | 0 | China |
| **(49)** | Wan 2020 | Antibody tests (IgG) | 6 | 0 | 1 | 10 | Singapore |
| **(50)** | Wang Q 2020 | Antibody tests (IgG) | 14 | 0 | 22 | 50 | China |
| **(51)** | Xiang J 2020 [1] | Antibody tests (IgG) | 52 | 0 | 11 | 35 | China |
| **(51)** | Xiang J 2020 [2] | Antibody tests (IgG) | 74 | 0 | 17 | 35 | China |
| **(52)** | Xiang F 2020 | Antibody tests (IgG) | 72 | 3 | 18 | 57 | China |
| **(53)** | Xiao DA 2020 | Antibody tests (IgG) | 32 | 0 | 2 | 0 | China |
| **(54)** | Xie J 2020 | Antibody tests (IgG) | 56 | 0 | 0 | 0 | China |
| **(55)** | Xu Y 2020 | Antibody tests (IgG) | 3 | 0 | 7 | 0 | China |
| **(56)** | Yang 2020 [1] | Antibody tests (IgG) | 34 | 0 | 36 | 0 | USA |
| **(56)** | Yang 2020 [2] | Antibody tests (IgG) | 60 | 0 | 10 | 0 | USA |
| **(56)** | Yang 2020 [3] | Antibody tests (IgG) | 70 | 0 | 0 | 0 | USA |
| **(57)** | Zeng Z 2020 | Antibody tests (IgG) | 0 | 0 | 27 | 36 | China |
| **(58)** | Zhang B 2020 | Antibody tests (IgG) | 219 | 0 | 3 | 0 | China |
| **(59)** | Zhang J 2020 | Antibody tests (IgG) | 3 | 1 | 0 | 224 | China |
| **(60)** | Zhang W 2020 | Antibody tests (IgG) | 13 | 0 | 3 | 0 | China |
| **(61)** | Zhao Z 2020 | Antibody tests (IgG) | 112 | 2 | 61 | 195 | China |
| **(62)** | Zhong 2020 [1] | Antibody tests (IgG) | 46 | 1 | 1 | 299 | China |
| **(62)** | Zhong 2020 [2] | Antibody tests (IgG) | 45 | 43 | 2 | 257 | China |
| **(62)** | Zhong 2020 [3] | Antibody tests (IgG) | 45 | 10 | 2 | 290 | China |
| **(19)** | Adams 2020 [1] | Antibody tests (IgM) | 28 | 0 | 12 | 50 | UK |
| **(19)** | Adams 2020 [2] | Antibody tests (IgM) | 3 | 0 | 30 | 60 | UK |
| **(19)** | Adams 2020 [3] | Antibody tests (IgM) | 2 | 0 | 36 | 60 | UK |
| **(19)** | Adams 2020 [4] | Antibody tests (IgM) | 15 | 1 | 16 | 59 | UK |
| **(19)** | Adams 2020 [5] | Antibody tests (IgM) | 14 | 1 | 17 | 59 | UK |
| **(19)** | Adams 2020 [6] | Antibody tests (IgM) | 17 | 1 | 16 | 59 | UK |
| **(19)** | Adams 2020 [7] | Antibody tests (IgM) | 14 | 0 | 18 | 60 | UK |
| **(19)** | Adams 2020 [8] | Antibody tests (IgM) | 15 | 4 | 25 | 138 | UK |
| **(20)** | Cai X 2020 | Antibody tests (IgM) | 158 | 0 | 118 | 167 | China |
| **(21)** | Cassaniti 2020 [1] | Antibody tests (IgM) | 25 | 0 | 5 | 30 | Italy |
| **(21)** | Cassaniti 2020 [2] | Antibody tests (IgM) | 6 | 1 | 32 | 11 | Italy |
| **(23)** | Du 2020 | Antibody tests (IgM) | 47 | 0 | 13 | 0 | China |
| **(24)** | Freeman 2020 | Antibody tests (IgM) | 75 | 0 | 24 | 0 | USA |
| **(25)** | Gao Y 2020 | Antibody tests (IgM) | 19 | 0 | 19 | 0 | China |
| **(26)** | Gao HX 2020 [1] | Antibody tests (IgM) | 14 | 0 | 23 | 0 | China |
| **(26)** | Gao HX 2020 [2] | Antibody tests (IgM) | 19 | 0 | 18 | 0 | China |
| **(26)** | Gao HX 2020 [3] | Antibody tests (IgM) | 11 | 0 | 26 | 0 | China |
| **(27)** | Garcia 2020 [1] | Antibody tests (IgM) | 12 | 0 | 43 | 45 | Spain |
| **(27)** | Garcia 2020 [2] | Antibody tests (IgM) | 25 | 0 | 38 | 0 | Spain |
| **(29)** | Guo L 2020 | Antibody tests (IgM) | 188 | 0 | 20 | 135 | China |
| **(30)** | Infantino 2020 | Antibody tests (IgM) | 46 | 4 | 15 | 59 | Italy |
| **(31)** | Jia 2020 | Antibody tests (IgM) | 39 | 0 | 18 | 0 | China |
| **(32)** | Jin 2020 | Antibody tests (IgM) | 13 | 0 | 14 | 33 | China |
| **(34)** | Li Z 2020 | Antibody tests (IgM) | 328 | 0 | 69 | 0 | China |
| **(35)** | Lin D 2020 [1] | Antibody tests (IgM) | 65 | 15 | 14 | 65 | China |
| **(35)** | Lin D 2020 [2] | Antibody tests (IgM) | 30 | 14 | 35 | 50 | China |
| **(36)** | Lippi 2020 [1] | Antibody tests (IgM) | 6 | 0 | 42 | 0 | Italy |
| **(37)** | Liu Y 2020 | Antibody tests (IgM) | 35 | 5 | 60 | 79 | China |
| **(38)** | Liu L 2020 | Antibody tests (IgM) | 167 | 3 | 71 | 117 | China |
| **(39)** | Liu R 2020 | Antibody tests (IgM) | 105 | 0 | 28 | 0 | China |
| **(40)** | Liu W 2020 [1] | Antibody tests (IgM) | 146 | 0 | 68 | 100 | China |
| **(40)** | Liu W 2020 [2] | Antibody tests (IgM) | 165 | 0 | 49 | 100 | China |
| **(41)** | Long 2020 | Antibody tests (IgM) | 243 | 0 | 120 | 0 | China |
| **(42)** | Lou 2020 [1] | Antibody tests (IgM) | 74 | 0 | 6 | 300 | China |
| **(42)** | Lou 2020 [2] | Antibody tests (IgM) | 71 | 4 | 9 | 205 | China |
| **(42)** | Lou 2020 [3] | Antibody tests (IgM) | 69 | 2 | 11 | 298 | China |
| **(43)** | Ma H 2020 | Antibody tests (IgM) | 209 | 37 | 7 | 446 | China |
| **(45)** | Padoan 2020 | Antibody tests (IgM) | 44 | 0 | 26 | 0 | Italy |
| **(46)** | Pan Y 2020 | Antibody tests (IgM) | 56 | 0 | 52 | 0 | China |
| **(47)** | Qian 2020 | Antibody tests (IgM) | 470 | 29 | 85 | 1529 | China |
| **(48)** | To KK 2020 [1] | Antibody tests (IgM) | 14 | 0 | 2 | 0 | China |
| **(48)** | To KK 2020 [2] | Antibody tests (IgM) | 15 | 0 | 1 | 0 | China |
| **(49)** | Wan 2020 | Antibody tests (IgM) | 2 | 1 | 5 | 9 | Singapore |
| **(50)** | Wang Q 2020 | Antibody tests (IgM) | 14 | 22 | 0 | 50 | China |
| **(51)** | Xiang J 2020 [1] | Antibody tests (IgM) | 35 | 0 | 28 | 35 | China |
| **(51)** | Xiang J 2020 [2] | Antibody tests (IgM) | 52 | 0 | 39 | 35 | China |
| **(52)** | Xiang F 2020 | Antibody tests (IgM) | 72 | 0 | 18 | 60 | China |
| **(53)** | Xiao DA 2020 | Antibody tests (IgM) | 28 | 0 | 6 | 0 | China |
| **(54)** | Xie J 2020 | Antibody tests (IgM) | 49 | 0 | 7 | 0 | China |
| **(55)** | Xu Y 2020 | Antibody tests (IgM) | 4 | 0 | 6 | 0 | China |
| **(56)** | Yang 2020 [1] | Antibody tests (IgM) | 38 | 0 | 32 | 0 | USA |
| **(56)** | Yang 2020 [2] | Antibody tests (IgM) | 60 | 0 | 10 | 0 | USA |
| **(56)** | Yang 2020 [3] | Antibody tests (IgM) | 70 | 0 | 0 | 0 | USA |
| **(57)** | Zeng Z 2020 | Antibody tests (IgM) | 0 | 0 | 27 | 36 | China |
| **(58)** | Zhang B 2020 | Antibody tests (IgM) | 182 | 0 | 40 | 0 | China |
| **(59)** | Zhang J 2020 | Antibody tests (IgM) | 3 | 6 | 0 | 219 | China |
| **(60)** | Zhang W 2020 | Antibody tests (IgM) | 16 | 0 | 0 | 0 | China |
| **(61)** | Zhao Z 2020 | Antibody tests (IgM) | 143 | 3 | 30 | 210 | China |
| **(62)** | Zhong 2020 [1] | Antibody tests (IgM) | 46 | 1 | 1 | 299 | China |
| **(62)** | Zhong 2020 [2] | Antibody tests (IgM) | 42 | 9 | 5 | 291 | China |
| **(62)** | Zhong 2020 [3] | Antibody tests (IgM) | 46 | 14 | 1 | 286 | China |

Antibodies immunoglobulin G and M (IgG and IgM); TP, true positive; FP, false positive; FN, false negative; TN, true negative.

**References:**

1. D. Kundu, P. Gautam, D. Dayanand, K. Gunasekaran, A. Manesh, M. Sebastian, K. P. P. Abhilash, A. Zachariah, T. George, S. Sathyendra, S. G. Hansdak, O. C. Abraham, R. Iyadurai, B. Thangakunam, R. Gupta, R. Karthik, M. Moorthy and G. M. Varghese: The role and diagnostic accuracy of serology for COVID-19. BMC Infect Dis, 22(1), 390 (2022) doi:10.1186/s12879-022-07361-y

2. M. T. Haghi Ashtiani, P. Sadeghi Rad, K. Asnaashari, A. Shahhosseini, F. Berenji and S. Mamishi: Role of serology tests in COVID-19 non-hospitalized patients: A cross-sectional study. PLoS One, 17(4), e0266923 (2022) doi:10.1371/journal.pone.0266923

3. F. Buntinx, P. Claes, M. Gulikers, J. Verbakel, L. Jan, M. Van der Elst, J. Van Elslande, M. Van Ranst and P. Vermeersch: Added value of anti-SARS-CoV-2 antibody testing in a Flemish nursing home during an acute COVID-19 outbreak in April 2020. Acta Clin Belg, 77(2), 295-300 (2022) doi:10.1080/17843286.2020.1834285

4. S. Chamkhi, T. Dhaouadi, I. Sfar, S. Mokni, A. Jebri, D. Mansouri, S. Ghedira, E. Ben Jemia, S. Ben Boujemaa, M. Houissa, H. Aouina, T. Ben Abdallah and Y. Gorgi: Comparative study of six SARS-CoV-2 serology assays: Diagnostic performance and antibody dynamics in a cohort of hospitalized patients for moderate to critical COVID-19. Int J Immunopathol Pharmacol, 36, 20587384211073232 (2022) doi:10.1177/20587384211073232

5. A. Abdollahi, S. Salarvand, V. Mehrtash, B. Jafarzadeh, M. Salehi, R. Ghalehtaki and S. Nateghi: The Performance of SARS-CoV-2 Serology Testing in the Diagnosis of COVID-19. Iran J Pathol, 17(1), 65-70 (2022) doi:10.30699/ijp.2021.526032.25971

6. A. Ozturk, T. Bozok and T. Simsek Bozok: Evaluation of rapid antibody test and chest computed tomography results of COVID-19 patients: A retrospective study. J Med Virol, 93(12), 6582-6587 (2021) doi:10.1002/jmv.27209

7. E. Diani, P. P. Piccaluga, V. Lotti, A. Di Clemente, M. Ligozzi, P. De Nardo, L. Lambertenghi, F. Pizzolo, S. Friso, G. Lo Cascio, A. Vianello, G. Marchi, E. Concia and D. Gibellini: Assessment of SARS-CoV-2 IgG and IgM antibody detection with a lateral flow immunoassay test. Heliyon, 7(10), e08192 (2021) doi:10.1016/j.heliyon.2021.e08192

8. I. Kiziloglu, A. Sener and N. Siliv: Comparison of rapid antibody test and thorax computed tomography results in patients who underwent RT-PCR with the pre-diagnosis of COVID-19. Int J Clin Pract, 75(10), e14524 (2021) doi:10.1111/ijcp.14524

9. Y. C. Lin, Y. L. Lee, C. Y. Cheng, W. P. Tseng, J. L. Wu, C. H. Lin, M. Y. Chung, C. M. Kang, Y. F. Lee, C. P. Chen, C. H. Huang, C. E. Liu, S. H. Cheng, S. C. Chen, S. Y. Chen and P. R. Hsueh: Multicenter evaluation of four immunoassays for the performance of early diagnosis of COVID-19 and assessment of antibody responses of patients with pneumonia in Taiwan. J Microbiol Immunol Infect, 54(5), 816-829 (2021) doi:10.1016/j.jmii.2021.02.003

10. I. Sekirov, V. E. Barakauskas, J. Simons, D. Cook, B. Bates, L. Burns, S. Masud, M. Charles, M. McLennan, A. Mak, N. Chahil, R. Vijh, A. Hayden, D. Goldfarb, P. N. Levett, M. Krajden and M. Morshed: SARS-CoV-2 serology: Validation of high-throughput chemiluminescent immunoassay (CLIA) platforms and a field study in British Columbia. J Clin Virol, 142, 104914 (2021) doi:10.1016/j.jcv.2021.104914

11. B. Poore, R. D. Nerenz, D. Brodis, C. I. Brown, M. A. Cervinski and J. A. Hubbard: A comparison of SARS-CoV-2 nucleocapsid and spike antibody detection using three commercially available automated immunoassays. Clin Biochem, 95, 77-80 (2021) doi:10.1016/j.clinbiochem.2021.05.011

12. E. English, L. E. Cook, I. Piec, S. Dervisevic, W. D. Fraser and W. G. John: Performance of the Abbott SARS-CoV-2 IgG II Quantitative Antibody Assay Including the New Variants of Concern, VOC 202012/V1 (United Kingdom) and VOC 202012/V2 (South Africa), and First Steps towards Global Harmonization of COVID-19 Antibody Methods. J Clin Microbiol, 59(9), e0028821 (2021) doi:10.1128/JCM.00288-21

13. S. Basgalupp, G. Dos Santos, M. Bessel, L. Garcia, A. C. de Moura, A. C. Rocha, E. Brito, G. de Miranda, T. Dornelles, W. Dartora, L. Pellanda, P. Hallal and E. Wendland: Diagnostic Properties of Three SARS-CoV-2 Antibody Tests. Diagnostics (Basel), 11(8) (2021) doi:10.3390/diagnostics11081441

14. K. Kubota, Y. Kitagawa, M. Matsuoka, K. Imai, Y. Orihara, R. Kawamura, J. Sakai, N. Ishibashi, N. Tarumoto, S. Takeuchi, S. Maesaki and T. Maeda: Clinical evaluation of the antibody response in patients with COVID-19 using automated high-throughput immunoassays. Diagn Microbiol Infect Dis, 100(3), 115370 (2021) doi:10.1016/j.diagmicrobio.2021.115370

15. D. Kim, J. Lee, J. Bal, C. K. Chong, J. H. Lee and H. Park: Clinical Evaluation of an Immunochromatographic-Based IgM/IgG Antibody Assay (GenBody COVI040) for Detection of Antibody Seroconversion in Patients with SARS-CoV-2 Infection. Diagnostics (Basel), 11(3) (2021) doi:10.3390/diagnostics11030537

16. S. Younes, H. Al-Jighefee, F. Shurrab, D. W. Al-Sadeq, N. Younes, S. R. Dargham, N. Al-Dewik, H. Qotba, M. Syed, A. Alnuaimi, H. M. Yassine, P. Tang, L. J. Abu-Raddad and G. K. Nasrallah: Diagnostic Efficiency of Three Fully Automated Serology Assays and Their Correlation with a Novel Surrogate Virus Neutralization Test in Symptomatic and Asymptomatic SARS-COV-2 Individuals. Microorganisms, 9(2) (2021) doi:10.3390/microorganisms9020245

17. Y. Boum, K. N. Fai, B. Nikolay, A. B. Mboringong, L. M. Bebell, M. Ndifon, A. Abbah, R. Essaka, L. Eteki, F. Luquero, C. Langendorf, N. F. Mbarga, R. G. Essomba, B. D. Buri, T. M. Corine, B. T. Kameni, N. Mandeng, M. Fanne, A.-C. Z.-K. Bisseck, C. B. Ndongmo, S. Eyangoh, A. Hamadou, J. P. Ouamba, M. T. Koku, R. Njouom, O. M. Claire, L. Esso, E. Epée and G. A. E. Mballa: Performance and operational feasibility of antigen and antibody rapid diagnostic tests for COVID-19 in symptomatic and asymptomatic patients in Cameroon: a clinical, prospective, diagnostic accuracy study. The Lancet Infectious Diseases, 21(8), 1089-1096 (2021) doi:10.1016/s1473-3099(21)00132-8

18. L. Cs, H. Sp, L. Yl, P. Sk and A. Tc: Performance of an automated chemiluminescent immunoassay for SARS-COV-2 IgM and head-to-head comparison of Abbott and Roche COVID-19 antibody assays. Pract Lab Med, 25, e00230 (2021) doi:10.1016/j.plabm.2021.e00230

19. E. R. Adams, M. Ainsworth, R. Anand, M. I. Andersson, K. Auckland, J. K. Baillie, E. Barnes, S. Beer, J. I. Bell, T. Berry, S. Bibi, M. Carroll, S. K. Chinnakannan, E. Clutterbuck, R. J. Cornall, D. W. Crook, T. de Silva, W. Dejnirattisai, K. E. Dingle, C. Dold, A. Espinosa, D. W. Eyre, H. Farmer, M. Fernandez Mendoza, D. Georgiou, S. J. Hoosdally, A. Hunter, K. Jefferey, D. F. Kelly, P. Klenerman, J. Knight, C. Knowles, A. J. Kwok, U. Leuschner, R. Levin, C. Liu, C. Lopez-Camacho, J. Martinez, P. C. Matthews, H. McGivern, A. J. Mentzer, J. Milton, J. Mongkolsapaya, S. C. Moore, M. S. Oliveira, F. Pereira, E. Perez, T. Peto, R. J. Ploeg, A. Pollard, T. Prince, D. J. Roberts, J. K. Rudkin, V. Sanchez, G. R. Screaton, M. G. Semple, J. Slon-Campos, D. T. Skelly, E. N. Smith, A. Sobrinodiaz, J. Staves, D. I. Stuart, P. Supasa, T. Surik, H. Thraves, P. Tsang, L. Turtle, A. S. Walker, B. Wang, C. Washington, N. Watkins, J. Whitehouse and C. T. S. A. P. National: Antibody testing for COVID-19: A report from the National COVID Scientific Advisory Panel. Wellcome Open Res, 5, 139 (2020) doi:10.12688/wellcomeopenres.15927.1.

20. X. F. Cai, J. Chen, J. Li Hu, Q. X. Long, H. J. Deng, P. Liu, K. Fan, P. Liao, B. Z. Liu, G. C. Wu, Y. K. Chen, Z. J. Li, K. Wang, X. L. Zhang, W. G. Tian, J. L. Xiang, H. X. Du, J. Wang, Y. Hu, N. Tang, Y. Lin, J. H. Ren, L. Y. Huang, J. Wei, C. Y. Gan, Y. M. Chen, Q. Z. Gao, A. M. Chen, C. L. He, D. X. Wang, P. Hu, F. C. Zhou, A. L. Huang and D. Q. Wang: A Peptide-Based Magnetic Chemiluminescence Enzyme Immunoassay for Serological Diagnosis of Coronavirus Disease 2019. J Infect Dis, 222(2), 189-193 (2020) doi:10.1093/infdis/jiaa243

21. I. Cassaniti, F. Novazzi, F. Giardina, F. Salinaro, M. Sachs, S. Perlini, R. Bruno, F. Mojoli, F. Baldanti and C.-T. F. Members of the San Matteo Pavia: Performance of VivaDiag COVID-19 IgM/IgG Rapid Test is inadequate for diagnosis of COVID-19 in acute patients referring to emergency room department. J Med Virol, 92(10), 1724-1727 (2020) doi:10.1002/jmv.25800

22. Z. Chen, Z. Zhang, X. Zhai, Y. Li, L. Lin, H. Zhao, L. Bian, P. Li, L. Yu, Y. Wu and G. Lin: Rapid and Sensitive Detection of anti-SARS-CoV-2 IgG, Using Lanthanide-Doped Nanoparticles-Based Lateral Flow Immunoassay. Anal Chem, 92(10), 7226-7231 (2020) doi:10.1021/acs.analchem.0c00784

23. Z. Du, F. Zhu, F. Guo, B. Yang and T. Wang: Detection of antibodies against SARS-CoV-2 in patients with COVID-19. J Med Virol, 92(10), 1735-1738 (2020) doi:10.1002/jmv.25820

24. B. Freeman, S. Lester, L. Mills, M. A. U. Rasheed, S. Moye, O. Abiona, G. B. Hutchinson, M. Morales-Betoulle, I. Krapinunaya, A. Gibbons, C. F. Chiang, D. Cannon, J. Klena, J. A. Johnson, S. M. Owen, B. S. Graham, K. S. Corbett and N. J. Thornburg: Validation of a SARS-CoV-2 spike protein ELISA for use in contact investigations and serosurveillance. bioRxiv (2020) doi:10.1101/2020.04.24.057323

25. G. Yong, Y. Yi, L. Tuantuan, W. Xiaowu, L. Xiuyong, L. Ang and H. Mingfeng: Evaluation of the auxiliary diagnostic value of antibody assays for the detection of novel coronavirus (SARS-CoV-2). J Med Virol, 92(10), 1975-1979 (2020) doi:10.1002/jmv.25919

26. H. X. Gao, Y. N. Li, Z. G. Xu, Y. L. Wang, H. B. Wang, J. F. Cao, D. Q. Yuan, L. Li, Y. Xu, Z. Zhang, Y. Huang, J. H. Lu, Y. Z. Liu and E. H. Dai: Detection of serum immunoglobulin M and immunoglobulin G antibodies in 2019 novel coronavirus infected patients from different stages. Chin Med J (Engl), 133(12), 1479-1480 (2020) doi:10.1097/CM9.0000000000000820

27. F. Pérez-García, R. Pérez-Tanoira, J. Romanyk, T. Arroyo, P. Gómez-Herruz and J. Cuadros-González: Rapid diagnosis of SARS-CoV-2 infection by detecting IgG and IgM antibodies with an immunochromatographic device: a prospective single-center study. medRxiv, 2020.04.11.20062158 (2020) doi:10.1101/2020.04.11.20062158

28. L. Grzelak, S. Temmam, C. Planchais, C. Demeret, L. Tondeur, C. Huon, F. Guivel-Benhassine, I. Staropoli, M. Chazal, J. Dufloo, D. Planas, J. Buchrieser, M. M. Rajah, R. Robinot, F. Porrot, M. Albert, K. Y. Chen, B. Crescenzo-Chaigne, F. Donati, F. Anna, P. Souque, M. Gransagne, J. Bellalou, M. Nowakowski, M. Backovic, L. Bouadma, L. Le Fevre, Q. Le Hingrat, D. Descamps, A. Pourbaix, C. Laouenan, J. Ghosn, Y. Yazdanpanah, C. Besombes, N. Jolly, S. Pellerin-Fernandes, O. Cheny, M. N. Ungeheuer, G. Mellon, P. Morel, S. Rolland, F. A. Rey, S. Behillil, V. Enouf, A. Lemaitre, M. A. Creach, S. Petres, N. Escriou, P. Charneau, A. Fontanet, B. Hoen, T. Bruel, M. Eloit, H. Mouquet, O. Schwartz and S. van der Werf: A comparison of four serological assays for detecting anti-SARS-CoV-2 antibodies in human serum samples from different populations. Sci Transl Med, 12(559) (2020) doi:10.1126/scitranslmed.abc3103

29. L. Guo, L. Ren, S. Yang, M. Xiao, Chang, F. Yang, C. S. Dela Cruz, Y. Wang, C. Wu, Y. Xiao, L. Zhang, L. Han, S. Dang, Y. Xu, Q. W. Yang, S. Y. Xu, H. D. Zhu, Y. C. Xu, Q. Jin, L. Sharma, L. Wang and J. Wang: Profiling Early Humoral Response to Diagnose Novel Coronavirus Disease (COVID-19). Clin Infect Dis, 71(15), 778-785 (2020) doi:10.1093/cid/ciaa310

30. M. Infantino, V. Grossi, B. Lari, R. Bambi, A. Perri, M. Manneschi, G. Terenzi, I. Liotti, G. Ciotta, C. Taddei, M. Benucci, P. Casprini, F. Veneziani, S. Fabbri, A. Pompetti and M. Manfredi: Diagnostic accuracy of an automated chemiluminescent immunoassay for anti-SARS-CoV-2 IgM and IgG antibodies: an Italian experience. J Med Virol, 92(9), 1671-1675 (2020) doi:10.1002/jmv.25932

31. X. Jia, P. Zhang, Y. Tian, J. Wang, H. Zeng, J. Wang, J. Liu, Z. Chen, L. Zhang, H. He, K. He and Y. Liu: Clinical Significance of an IgM and IgG Test for Diagnosis of Highly Suspected COVID-19. Front Med (Lausanne), 8, 569266 (2021) doi:10.3389/fmed.2021.569266

32. Y. Jin, M. Wang, Z. Zuo, C. Fan, F. Ye, Z. Cai, Y. Wang, H. Cui, K. Pan and A. Xu: Diagnostic value and dynamic variance of serum antibody in coronavirus disease 2019. Int J Infect Dis, 94, 49-52 (2020) doi:10.1016/j.ijid.2020.03.065

33. R. Lassaunière, A. Frische, Z. B. Harboe, A. C. Y. Nielsen, A. Fomsgaard, K. A. Krogfelt and C. S. Jørgensen: Evaluation of nine commercial SARS-CoV-2 immunoassays. medRxiv, 2020.04.09.20056325 (2020) doi:10.1101/2020.04.09.20056325

34. Z. Li, Y. Yi, X. Luo, N. Xiong, Y. Liu, S. Li, R. Sun, Y. Wang, B. Hu, W. Chen, Y. Zhang, J. Wang, B. Huang, Y. Lin, J. Yang, W. Cai, X. Wang, J. Cheng, Z. Chen, K. Sun, W. Pan, Z. Zhan, L. Chen and F. Ye: Development and clinical application of a rapid IgM-IgG combined antibody test for SARS-CoV-2 infection diagnosis. J Med Virol, 92(9), 1518-1524 (2020) doi:10.1002/jmv.25727

35. D. Lin, L. Liu, M. Zhang, Y. Hu, Q. Yang, J. Guo, Y. Dai, Y. Xu, Y. Cai, X. Chen, K. Huang and Z. Zhang: Evaluations of the serological test in the diagnosis of 2019 novel coronavirus (SARS-CoV-2) infections during the COVID-19 outbreak. Eur J Clin Microbiol Infect Dis, 39(12), 2271-2277 (2020) doi:10.1007/s10096-020-03978-6

36. G. Lippi, G. L. Salvagno, M. Pegoraro, V. Militello, C. Caloi, A. Peretti, S. Gaino, A. Bassi, C. Bovo and G. Lo Cascio: Assessment of immune response to SARS-CoV-2 with fully automated MAGLUMI 2019-nCoV IgG and IgM chemiluminescence immunoassays. Clin Chem Lab Med, 58(7), 1156-1159 (2020) doi:10.1515/cclm-2020-0473

37. L. Ying, L. Yue-ping, D. Bo, R. Feifei, W. Yue, D. Jinya and H. Qianchuan: Diagnostic Indexes of a Rapid IgG/IgM Combined Antibody Test for SARS-CoV-2. medRxiv, 2020.03.26.20044883 (2020) doi:10.1101/2020.03.26.20044883

38. L. Liu, W. Liu, Y. Zheng, X. Jiang, G. Kou, J. Ding, Q. Wang, Q. Huang, Y. Ding, W. Ni, W. Wu, S. Tang, L. Tan, Z. Hu, W. Xu, Y. Zhang, B. Zhang, Z. Tang, X. Zhang, H. Li, Z. Rao, H. Jiang, X. Ren, S. Wang and S. Zheng: A preliminary study on serological assay for severe acute respiratory syndrome coronavirus 2 (SARS-CoV-2) in 238 admitted hospital patients. Microbes Infect, 22(4-5), 206-211 (2020) doi:10.1016/j.micinf.2020.05.008

39. R. Liu, X. Liu, H. Han, M. A. Shereen, Z. Niu, D. Li, F. Liu, K. Wu, Z. Luo and C. Zhu: The comparative superiority of IgM-IgG antibody test to real-time reverse transcriptase PCR detection for SARS-CoV-2 infection diagnosis. medRxiv, 2020.03.28.20045765 (2020) doi:10.1101/2020.03.28.20045765

40. W. Liu, L. Liu, G. Kou, Y. Zheng, Y. Ding, W. Ni, Q. Wang, L. Tan, W. Wu, S. Tang, Z. Xiong and S. Zheng: Evaluation of Nucleocapsid and Spike Protein-Based Enzyme-Linked Immunosorbent Assays for Detecting Antibodies against SARS-CoV-2. J Clin Microbiol, 58(6) (2020) doi:10.1128/JCM.00461-20

41. Q.-x. Long, H.-j. Deng, J. Chen, J.-l. Hu, B.-z. Liu, P. Liao, Y. Lin, L.-h. Yu, Z. Mo, Y.-y. Xu, F. Gong, G.-c. Wu, X.-x. Zhang, Y.-k. Chen, Z.-j. Li, K. Wang, X.-l. Zhang, W.-g. Tian, C.-c. Niu, Q.-j. Yang, J.-l. Xiang, H.-x. Du, H.-w. Liu, C.-h. Lang, X.-H. Luo, S.-b. Wu, X.-p. Cui, Z. Zhou, J. Wang, C.-j. Xue, X.-f. Li, L. Wang, X.-j. Tang, Y. Zhang, J.-f. Qiu, X.-m. Liu, J.-j. Li, D.-c. Zhang, F. Zhang, X.-f. Cai, D.-q. Wang, Y. Hu, J.-h. Ren, N. Tang, P. Liu, Q. Li and A.-l. Huang: Antibody responses to SARS-CoV-2 in COVID-19 patients: the perspective application of serological tests in clinical practice. medRxiv, 2020.03.18.20038018 (2020) doi:10.1101/2020.03.18.20038018

42. B. Lou, T. D. Li, S. F. Zheng, Y. Y. Su, Z. Y. Li, W. Liu, F. Yu, S. X. Ge, Q. D. Zou, Q. Yuan, S. Lin, C. M. Hong, X. Y. Yao, X. J. Zhang, D. H. Wu, G. L. Zhou, W. H. Hou, T. T. Li, Y. L. Zhang, S. Y. Zhang, J. Fan, J. Zhang, N. S. Xia and Y. Chen: Serology characteristics of SARS-CoV-2 infection after exposure and post-symptom onset. Eur Respir J, 56(2) (2020) doi:10.1183/13993003.00763-2020

43. H. Ma, W. Zeng, H. He, D. Zhao, D. Jiang, P. Zhou, L. Cheng, Y. Li, X. Ma and T. Jin: Serum IgA, IgM, and IgG responses in COVID-19. Cell Mol Immunol, 17(7), 773-775 (2020) doi:10.1038/s41423-020-0474-z

44. N. M. A. Okba, M. A. Muller, W. Li, C. Wang, C. H. GeurtsvanKessel, V. M. Corman, M. M. Lamers, R. S. Sikkema, E. de Bruin, F. D. Chandler, Y. Yazdanpanah, Q. Le Hingrat, D. Descamps, N. Houhou-Fidouh, C. Reusken, B. J. Bosch, C. Drosten, M. P. G. Koopmans and B. L. Haagmans: Severe Acute Respiratory Syndrome Coronavirus 2-Specific Antibody Responses in Coronavirus Disease Patients. Emerg Infect Dis, 26(7), 1478-1488 (2020) doi:10.3201/eid2607.200841

45. A. Padoan, C. Cosma, L. Sciacovelli, D. Faggian and M. Plebani: Analytical performances of a chemiluminescence immunoassay for SARS-CoV-2 IgM/IgG and antibody kinetics. Clin Chem Lab Med, 58(7), 1081-1088 (2020) doi:10.1515/cclm-2020-0443

46. Y. Pan, X. Li, G. Yang, J. Fan, Y. Tang, J. Zhao, X. Long, S. Guo, Z. Zhao, Y. Liu, H. Hu, H. Xue and Y. Li: Serological immunochromatographic approach in diagnosis with SARS-CoV-2 infected COVID-19 patients. J Infect, 81(1), e28-e32 (2020) doi:10.1016/j.jinf.2020.03.051

47. C. Qian, M. Zhou, F. Cheng, X. Lin, Y. Gong, X. Xie, P. Li, Z. Li, P. Zhang, Z. Liu, F. Hu, Y. Wang, Q. Li, Y. Zhu, G. Duan, Y. Xing, H. Song, W. Xu, B. F. Liu and F. Xia: Development and multicenter performance evaluation of fully automated SARS-CoV-2 IgM and IgG immunoassays. Clin Chem Lab Med, 58(9), 1601-1607 (2020) doi:10.1515/cclm-2020-0548

48. K. K.-W. To, O. T.-Y. Tsang, W.-S. Leung, A. R. Tam, T.-C. Wu, D. C. Lung, C. C.-Y. Yip, J.-P. Cai, J. M.-C. Chan, T. S.-H. Chik, D. P.-L. Lau, C. Y.-C. Choi, L.-L. Chen, W.-M. Chan, K.-H. Chan, J. D. Ip, A. C.-K. Ng, R. W.-S. Poon, C.-T. Luo, V. C.-C. Cheng, J. F.-W. Chan, I. F.-N. Hung, Z. Chen, H. Chen and K.-Y. Yuen: Temporal profiles of viral load in posterior oropharyngeal saliva samples and serum antibody responses during infection by SARS-CoV-2: an observational cohort study. The Lancet Infectious Diseases, 20(5), 565-574 (2020) doi:10.1016/s1473-3099(20)30196-1

49. W. Y. Wan, S. H. Lim and E. H. Seng: Cross-reaction of Sera from COVID-19 Patients with SARS-CoV Assays. Annals of the Academy of Medicine, Singapore, 49(7), 523-526 (2020) doi:10.47102/annals-acadmedsg.2020120

50. Q. Wang, Q. Du, B. Guo, D. Mu, X. Lu, Q. Ma, Y. Guo, L. Fang, B. Zhang, G. Zhang and X. Guo: A Method To Prevent SARS-CoV-2 IgM False Positives in Gold Immunochromatography and Enzyme-Linked Immunosorbent Assays. J Clin Microbiol, 58(6) (2020) doi:10.1128/JCM.00375-20

51. J. Xiang, M. Yan, H. Li, T. Liu, C. Lin, S. Huang and C. Shen: Evaluation of Enzyme-Linked Immunoassay and Colloidal Gold-Immunochromatographic Assay Kit for Detection of Novel Coronavirus (SARS-Cov-2) Causing an Outbreak of Pneumonia (COVID-19). medRxiv, 2020.02.27.20028787 (2020) doi:10.1101/2020.02.27.20028787

52. F. Xiang, X. Wang, X. He, Z. Peng, B. Yang, J. Zhang, Q. Zhou, H. Ye, Y. Ma, H. Li, X. Wei, P. Cai and W. L. Ma: Antibody Detection and Dynamic Characteristics in Patients With Coronavirus Disease 2019. Clin Infect Dis, 71(8), 1930-1934 (2020) doi:10.1093/cid/ciaa461

53. X. Fang, Q. Mei, T. Yang, L. Li, Y. Wang, F. Tong, S. Geng and A. Pan: Low-dose corticosteroid therapy does not delay viral clearance in patients with COVID-19. J Infect, 81(1), 147-178 (2020) doi:10.1016/j.jinf.2020.03.039

54. J. Xie, C. Ding, J. Li, Y. Wang, H. Guo, Z. Lu, J. Wang, C. Zheng, T. Jin, Y. Gao and H. He: Characteristics of patients with coronavirus disease (COVID-19) confirmed using an IgM-IgG antibody test. J Med Virol, 92(10), 2004-2010 (2020) doi:10.1002/jmv.25930

55. Y. Xu: Dynamic profile of severe or critical COVID-19 cases. medRxiv, 2020.03.18.20038513 (2020) doi:10.1101/2020.03.18.20038513

56. J. Yang, E. C. Pederson, C. Hamilton, T. Neibauer, K. Robyak, P. McGhee, T. Speicher and Y. Zhu: Analytical and Clinical Analysis of Two Automated Anti-SARS-CoV-2 Immunoassays in Pre-Pandemic and Pandemic Patient Populations. J Appl Lab Med, 6(2), 441-450 (2021) doi:10.1093/jalm/jfaa204

57. Z. Zeng, L. Chen, Y. Pan, Q. Deng, G. Ye, Y. Li and X. Wang: Re: Profile of specific antibodies to SARS-CoV-2: The first report. J Infect, 81(1), e80-e81 (2020) doi:10.1016/j.jinf.2020.03.052

58. B. Zhang, X. Zhou, C. Zhu, Y. Song, F. Feng, Y. Qiu, J. Feng, Q. Jia, Q. Song, B. Zhu and J. Wang: Immune Phenotyping Based on the Neutrophil-to-Lymphocyte Ratio and IgG Level Predicts Disease Severity and Outcome for Patients With COVID-19. Front Mol Biosci, 7, 157 (2020) doi:10.3389/fmolb.2020.00157

59. J. Zhang, X. Zhang, J. Liu, Y. Ban, N. Li, Y. Wu, Y. Liu, R. Ye, J. Liu, X. Li, L. Li, X. Qin and R. Zheng: Serological detection of 2019-nCoV respond to the epidemic: A useful complement to nucleic acid testing. Int Immunopharmacol, 88, 106861 (2020) doi:10.1016/j.intimp.2020.106861

60. W. Zhang, R. H. Du, B. Li, X. S. Zheng, X. L. Yang, B. Hu, Y. Y. Wang, G. F. Xiao, B. Yan, Z. L. Shi and P. Zhou: Molecular and serological investigation of 2019-nCoV infected patients: implication of multiple shedding routes. Emerg Microbes Infect, 9(1), 386-389 (2020) doi:10.1080/22221751.2020.1729071

61. J. Zhao, Q. Yuan, H. Wang, W. Liu, X. Liao, Y. Su, X. Wang, J. Yuan, T. Li, J. Li, S. Qian, C. Hong, F. Wang, Y. Liu, Z. Wang, Q. He, Z. Li, B. He, T. Zhang, Y. Fu, S. Ge, L. Liu, J. Zhang, N. Xia and Z. Zhang: Antibody Responses to SARS-CoV-2 in Patients With Novel Coronavirus Disease 2019. Clin Infect Dis, 71(16), 2027-2034 (2020) doi:10.1093/cid/ciaa344

62. L. Zhong, J. Chuan, B. Gong, P. Shuai, Y. Zhou, Y. Zhang, Z. Jiang, D. Zhang, X. Liu, S. Ma, Y. Huang, H. Lin, Q. Wang, L. Huang, D. Jiang, F. Hao, J. Tang, C. Zheng, H. Yu, Z. Wang, Q. Jiang, T. Zeng, M. Luo, F. Zeng, F. Zeng, J. Liu, J. Tian, Y. Xu, T. Long, K. Xu, X. Yang, Y. Liu, Y. Shi, L. Jiang and Z. Yang: Detection of serum IgM and IgG for COVID-19 diagnosis. Sci China Life Sci, 63(5), 777-780 (2020) doi:10.1007/s11427-020-1688-9

**Appendix 3.** Forest plot of likelihood ratios for positive test results of Ig G for predicting COVID-19 diagnosis


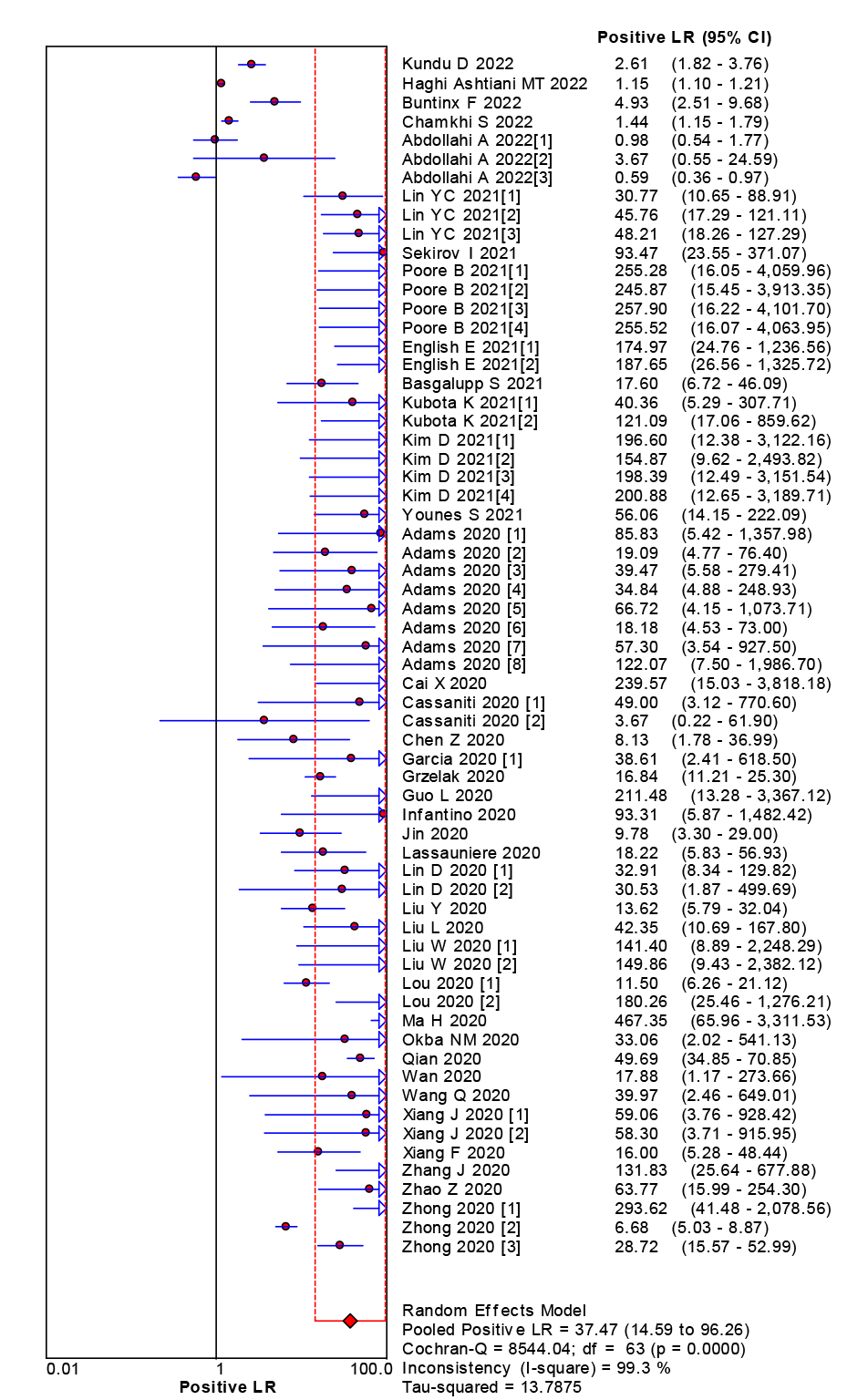


**Appendix 4.** Forest plot of likelihood ratios for negative test results of Ig G for predicting COVID-19 diagnosis


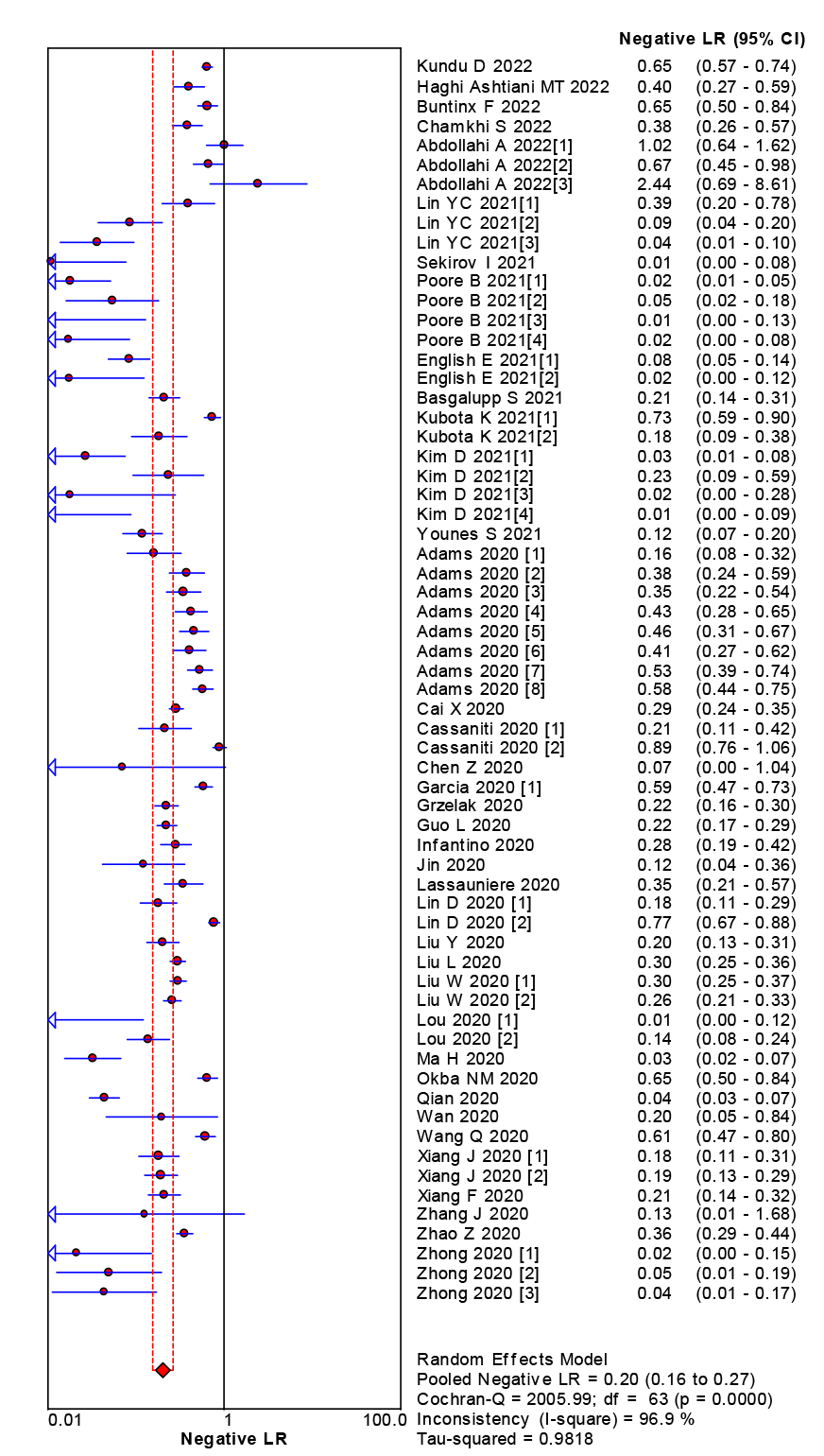


**Appendix 5.** Forest plot of likelihood ratios for positive test results of Ig M for predicting COVID-19 diagnosis


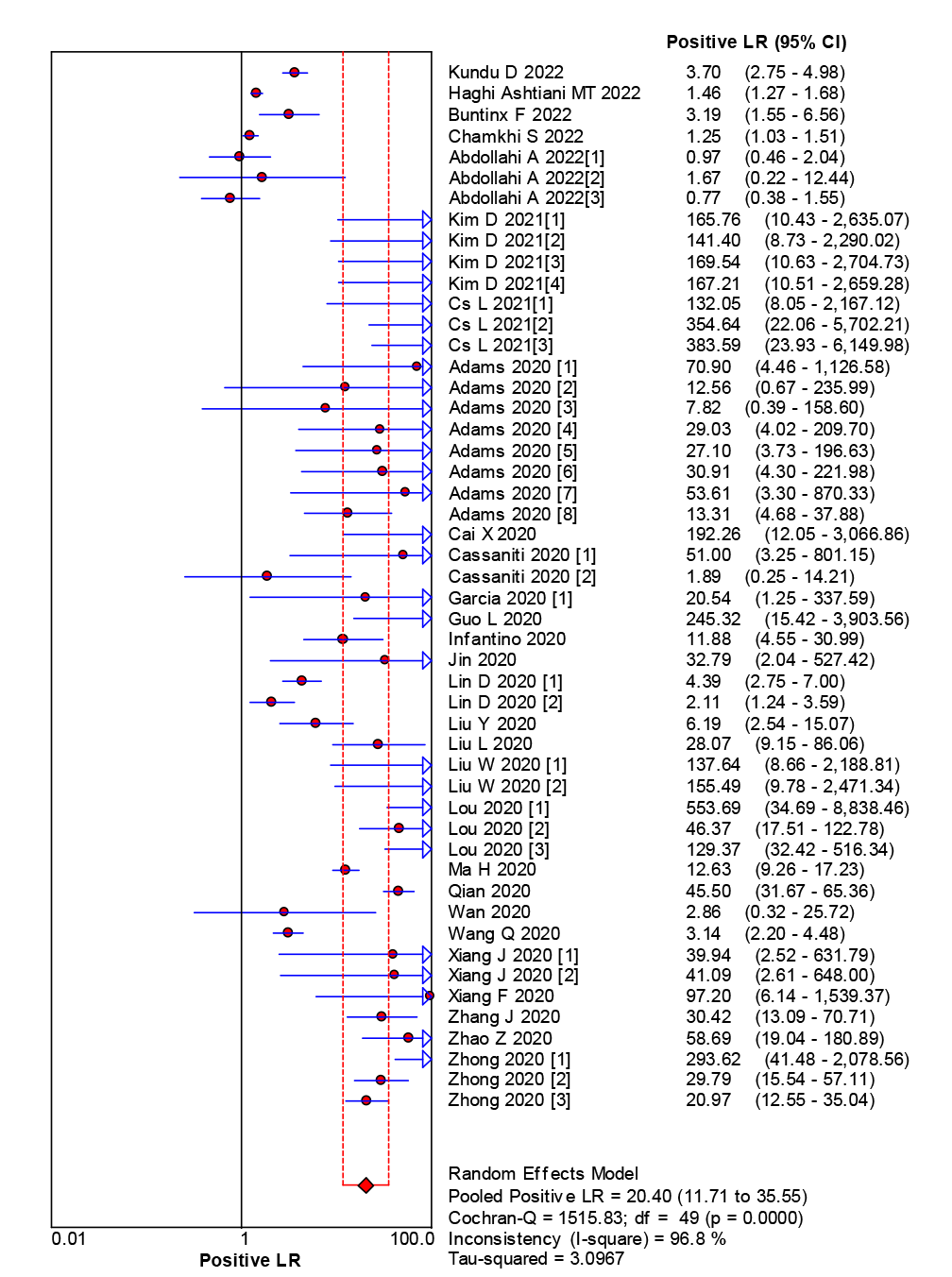


**Appendix 6.** Forest plot of likelihood ratios for negative test results of Ig M for predicting COVID-19 diagnosis


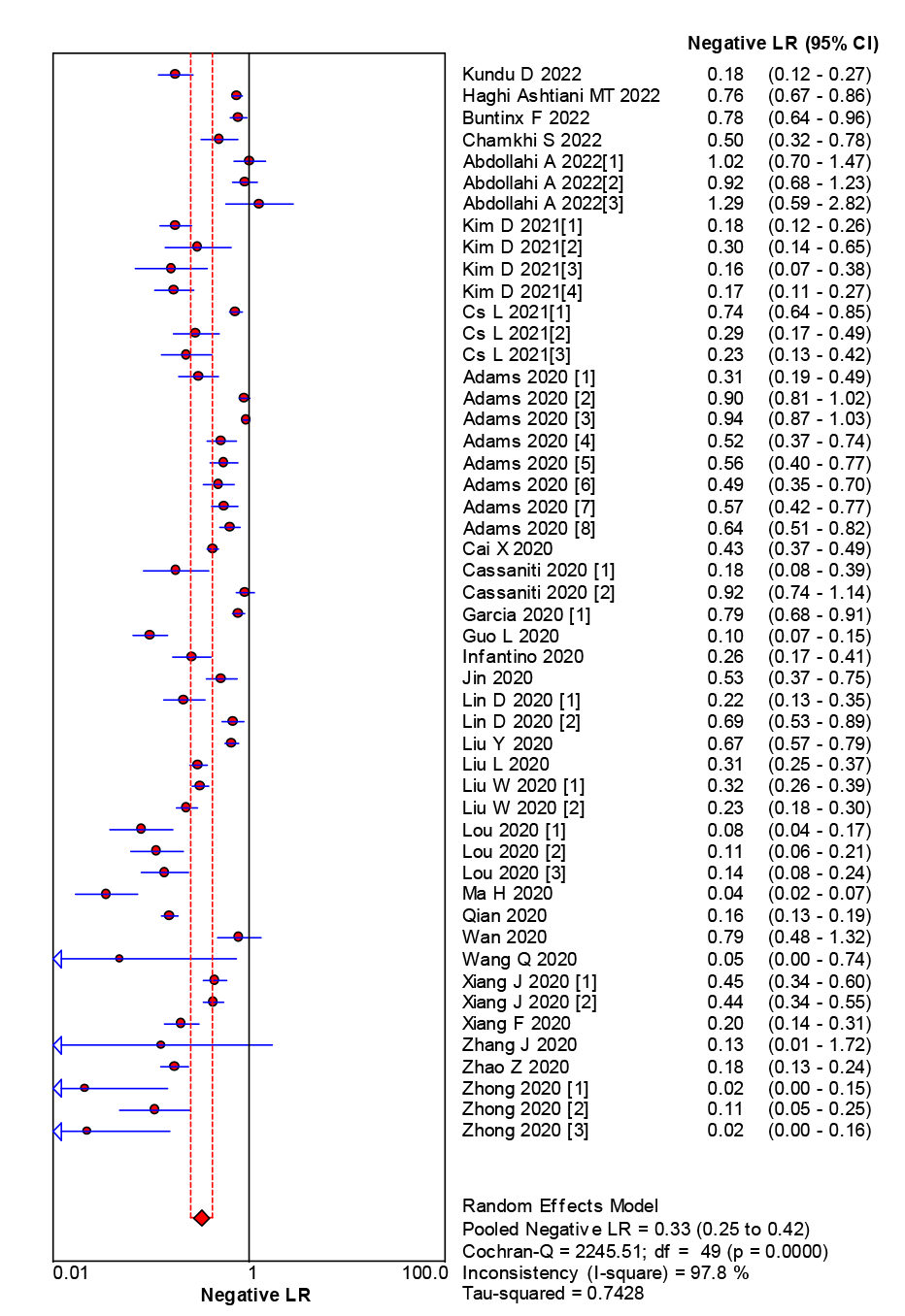

Supplement: Supplementary file 1 [file Data_Sheet_1.doc]
